# Activation of DAF-16/FOXO by reactive oxygen species contributes to longevity in long-lived mitochondrial mutants in *Caenorhabditis elegans*

**DOI:** 10.1371/journal.pgen.1007268

**Published:** 2018-03-09

**Authors:** Megan M. Senchuk, Dylan J. Dues, Claire E. Schaar, Benjamin K. Johnson, Zachary B. Madaj, Megan J. Bowman, Mary E. Winn, Jeremy M. Van Raamsdonk

**Affiliations:** 1 Laboratory of Aging and Neurodegenerative Disease (LAND), Center for Neurodegenerative Science, Van Andel Research Institute, Grand Rapids, Michigan, United States of America; 2 Bioinformatics and Biostatistics Core, Van Andel Research Institute, Grand Rapids, Michigan, United States of America; 3 Department of Neurology and Neurosurgery, McGill University, Montreal, Quebec, Canada; 4 Metabolic Disorders and Complications Program, and Brain Repair and Integrative Neuroscience Program, Research Institute of the McGill University Health Centre, Montreal, Quebec, Canada; ETH, SWITZERLAND

## Abstract

Mild deficits in mitochondrial function have been shown to increase lifespan in multiple species including worms, flies and mice. Here, we study three *C*. *elegans* mitochondrial mutants (*clk-1*, *isp-1* and *nuo-6*) to identify overlapping genetic pathways that contribute to their longevity. We find that genes regulated by the FOXO transcription factor DAF-16 are upregulated in all three strains, and that the transcriptional changes present in these worms overlap significantly with the long-lived insulin-IGF1 signaling pathway mutant *daf-2*. We show that DAF-16 and multiple DAF-16 interacting proteins (MATH-33, IMB-2, CST-1/2, BAR-1) are required for the full longevity of all three mitochondrial mutants. Our results suggest that the activation of DAF-16 in these mutants results from elevated levels of reactive oxygen species. Overall, this work reveals an overlapping genetic pathway required for longevity in three mitochondrial mutants, and, combined with previous work, demonstrates that DAF-16 is a downstream mediator of lifespan extension in multiple pathways of longevity.

## Author summary

The use of genetic model organisms has permitted the identification of a large number of genes that influence longevity. These genes have been grouped into different pathways of lifespan extension, which have been proposed to modulate longevity by distinct mechanisms. In this work, we explore the mechanisms underlying longevity in three long-lived mitochondrial mutants in *C*. *elegans*. We find that all three mutants show upregulation of DAF-16/FOXO target genes and that DAF-16 as well as multiple proteins that function with DAF-16 are required for their longevity. Since DAF-16 has previously been shown to be responsible for the increase in lifespan resulting from decreasing insulin-IGF1 signaling, this indicates that different pathways of lifespan extension have overlapping mechanisms, and that DAF-16/FOXO is a common downstream mediator of longevity.

## Introduction

Mitochondria serve a variety of critical functions within the cell including energy production, intracellular signaling and metabolism. Accordingly, major disruptions of mitochondrial function lead to detrimental effects including lethality. In contrast, mild disruption of mitochondrial function has been shown to increase lifespan.

The first demonstration that impairment of mitochondrial function could extend longevity came from the worm *C*. *elegans*. The gene *clk-1* was identified in a screen for maternal effect mutations that slow development and defecation cycle length. Mutations in *clk-1* were found to increase lifespan [1,2], and this gene was later found to encode a hydroxylase involved in the biosynthesis of ubiquinone [3]. This same screen identified nine other *clk* genes, all of which decrease mitochondrial function and increase lifespan [4]. Since then multiple other genes affecting mitochondrial function have been shown to increase lifespan in *C*. *elegans*, including *isp-1*, which encodes the Rieske iron-sulfur subunit of complex III of the mitochondrial electron transport chain [5], *nuo-6*, which encodes a subunit of complex I of the mitochondrial electron transport chain [6], *lrs-2*, which encodes a mitochondrial leucyl-tRNA synthetase [7], and *sod-2*, which encodes the primary mitochondrial superoxide dismutase [8]. In addition, an unbiased RNAi screen for genes that increase lifespan identified many genes involved in mitochondrial function [7].

The ability of decreasing mitochondrial function to extend longevity is not limited to *C*. *elegans*. As in worms, it has been shown that decreasing the expression of components of the mitochondrial electron transport chain in *Drosophila* increases lifespan [9]. Similarly, knocking down the expression of complex I subunit ND75 in *Drosophila* was also shown to extend longevity [10]. In mice, it has been shown that a mutation affecting the cytochrome c oxidase assembly factor SURF1 reduces mitochondrial function, but increases lifespan [11]. In addition, a heterozygous mutation in the mouse homolog of *clk-1*, *Mclk1*, causes mitochondrial dysfunction [12], and extended longevity [13]. Thus, the ability of a mild decrease in mitochondrial function to increase lifespan is conserved across species.

While a number of groups have studied the longevity resulting from impairment of mitochondrial function and identified factors involved [14–19], the mechanism by which decreasing mitochondrial function increases lifespan has not been fully elucidated. It is also uncertain to which extent the mechanism of lifespan extension is the same between different mitochondrial mutants, and the extent to which these mechanisms overlap with other pathways of lifespan extension.

One of the most well-studied pathways of lifespan extension is the insulin-IGF1 signaling pathway. The two first genes that were shown to increase lifespan in any species are part of this nutrient signaling pathway. Mutations of the insulin-IGF1 receptor *daf-2* [20], or other genes in the signaling pathway [21], can double the lifespan of the worm. This increase in lifespan is completely dependent on the FOXO transcription factor DAF-16 [20,22,23]. Mutations in the *daf-2* gene cause decreased insulin-IGF1 signaling, which leads to increased nuclear localization of DAF-16 [24] and altered expression of DAF-16 target genes [25–27]. At least some of these changes in gene expression are required for *daf-2* longevity [25].

In addition to its role in the insulin-IGF1 signaling pathway, DAF-16/FOXO has been shown to respond to various forms of stress. The nuclear localization of DAF-16 can be induced by heat stress, anoxia, oxidative stress, starvation and exposure to pathogenic bacteria [24,28]. As *daf-16* mutants show increased sensitivity to multiple types of stress [29–31], and overexpression of DAF-16 results in increased resistance to stress [24], it appears that DAF-16 also has an important role in stress resistance.

A number of previous studies have examined the interaction between mild impairment of mitochondrial function and the insulin-IGF1 signaling pathway using either genetic mitochondrial mutants [2,5], or RNAi against genes affecting mitochondrial function [7,32,33]. These studies have shown that loss of *daf-16* decreases the lifespan of long-lived worms with decreased mitochondrial function. However, these experiments also found that decreasing *daf-16* levels also shortens the lifespan of wild-type worms, making the role of *daf-16* in the longevity of the mitochondrial mutants and mitochondrial RNAi difficult to interpret without further experimentation.

In this work, we examine overlapping changes in gene expression in three long-lived mitochondrial mutant strains to identify common mediators of longevity. We find that all three strains show a modulation of DAF-16 target gene expression. Our results suggest that elevated levels of ROS in these mitochondrial mutants cause the activation and nuclear localization of DAF-16. Importantly, we find that DAF-16 and multiple DAF-16 interacting proteins are required for the full longevity of these mutants. This work suggests that there are converging downstream mechanisms contributing to lifespan extension between different mitochondrial mutants, and between long-lived mitochondrial mutants and other pathways of lifespan extension.

## Results

### Long-lived mitochondrial mutants have increased expression of DAF-16/FOXO target genes

**Fig 1:**
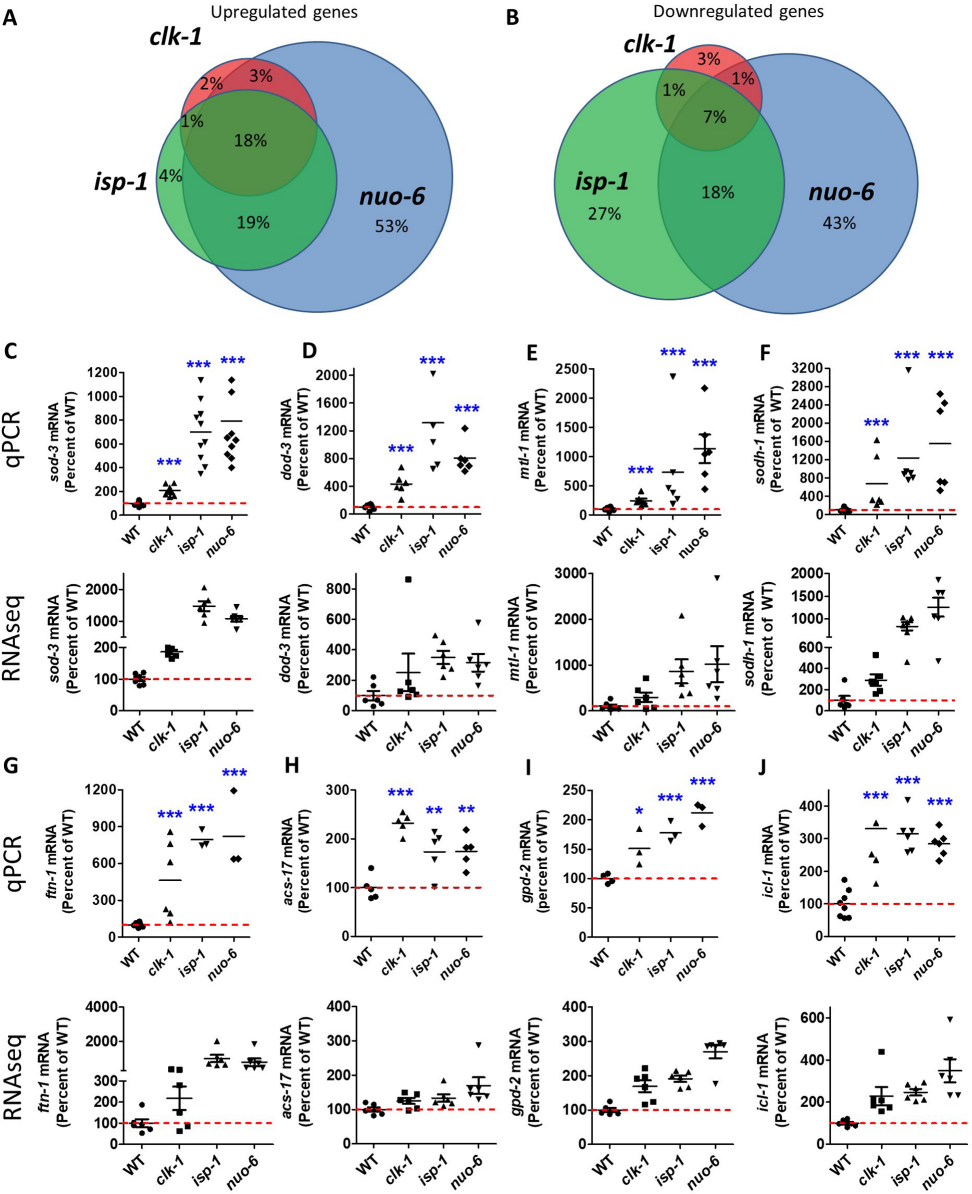
Long-lived mitochondrial mutants exhibit upregulation of DAF-16/FOXO target genes. Gene expression from three long-lived mitochondrial mutants (*clk-1*, *isp-1*, *nuo-6*) was compared to identify overlapping genetic pathways that contribute to longevity. **A.** Of all of the upregulated genes, 18% were in common between all three mutants and 40% in common between at least two of the mutants. Percentages indicate the percent out of the total number of genes upregulated in any of the three mutants. **B.** For downregulated genes, 7% were found in all three mitochondrial mutants and 27% were shared by at least two mitochondrial mutants. Percentages indicate the percent of all of the genes downregulated in the three mutants. A number of DAF-16 target genes were identified among the overlapping upregulated genes between the mitochondrial mutants. Quantitative real-time RT-PCR (qPCR) was used to confirm the upregulation of DAF-16 target genes in all three long-lived mitochondrial mutants including *sod-3* (**C**), *dod-3* (**D**), *mtl-1* (**E**), *sodh-1* (**F**), *ftn-1* (**G**), *acs-17* (**H**), *gpd-2* (**I**), and *icl-1* (**J**). For comparison individual values from RNAseq data are also shown below qPCR results. Error bars indicate SEM. *p<0.05, **p<0.01, ***p<0.001.

To identify overlapping genetic pathways that contribute to longevity in long-lived mitochondrial mutants, we compared gene expression in *clk-1*, *isp-1* and *nuo-6* worms to wild-type worms using RNA sequencing (RNAseq). RNA was isolated from six biological replicates per strain and sequenced individually. We observed a striking degree of overlap in the patterns of gene expression (see **S1 Table** for a complete list of significantly upregulated and downregulated genes). Of all of the genes upregulated in these strains, 18% were upregulated in all three strains, while 40% were upregulated in at least two strains (**Fig 1A**). Similarly, we found that 7% of all of the downregulated genes exhibited decreased expression in all three strains, and 27% were downregulated in at least two of the strains (**Fig 1B**). Of the genes that were commonly upregulated, we observed a number of known DAF-16 target genes including *sod-3*, *dod-3*, *mtl-1*, *sodh-1*, *ftn-1*, *gpd-2* and *icl-1*. Accordingly, we chose to focus the remainder of our study on the role of DAF-16 in the longevity of the long-lived mitochondrial mutants.

To confirm our observation that DAF-16 target genes are upregulated in long-lived mitochondrial mutants, we tested eight specific DAF-16 target genes using quantitative real-time RT-PCR. These genes were chosen from the list of the top DAF-16 target genes identified in a meta-analysis of previous DAF-16 gene expression studies by Tepper *et al*., 2013 [27]. As with the data from RNA sequencing, we found that all eight DAF-16 target genes were significantly upregulated in *clk-1*, *isp-1* and *nuo-6* mutants (**Fig 1C–1J**).

**Fig 2:**
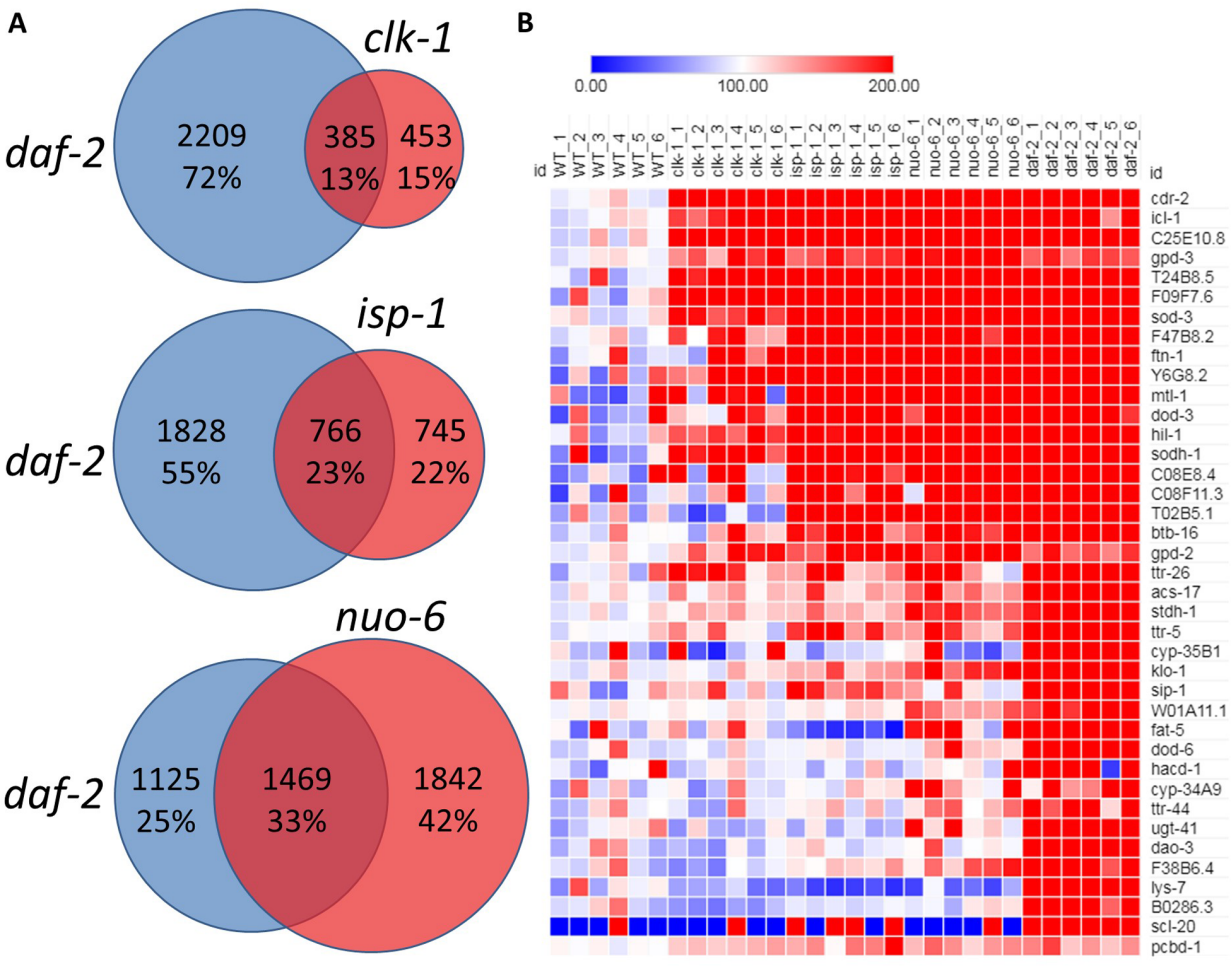
Overlap of upregulated genes between long-lived mitochondrial mutants and *daf-2* mutants. **A.** Among the genes upregulated in the long-lived mitochondrial mutant strains *clk-1*, *isp-1* and *nuo-6*, many of these genes (46%, 50% and 57% respectively) are also upregulated in *daf-2* mutants. **B.** A heat map showing the top DAF-16 responsive genes that are upregulated in *daf-2* mutants (genes included are from the meta-analysis of DAF-16 target genes performed by Tepper *et al*., 2013 that were significantly upregulated in our *daf-2* RNAseq data). Many of these genes are also upregulated in the long-lived mitochondrial mutant strains. mRNA for all strains was isolated from six biological replicates per strain and sequenced individually.

**Fig 3:**
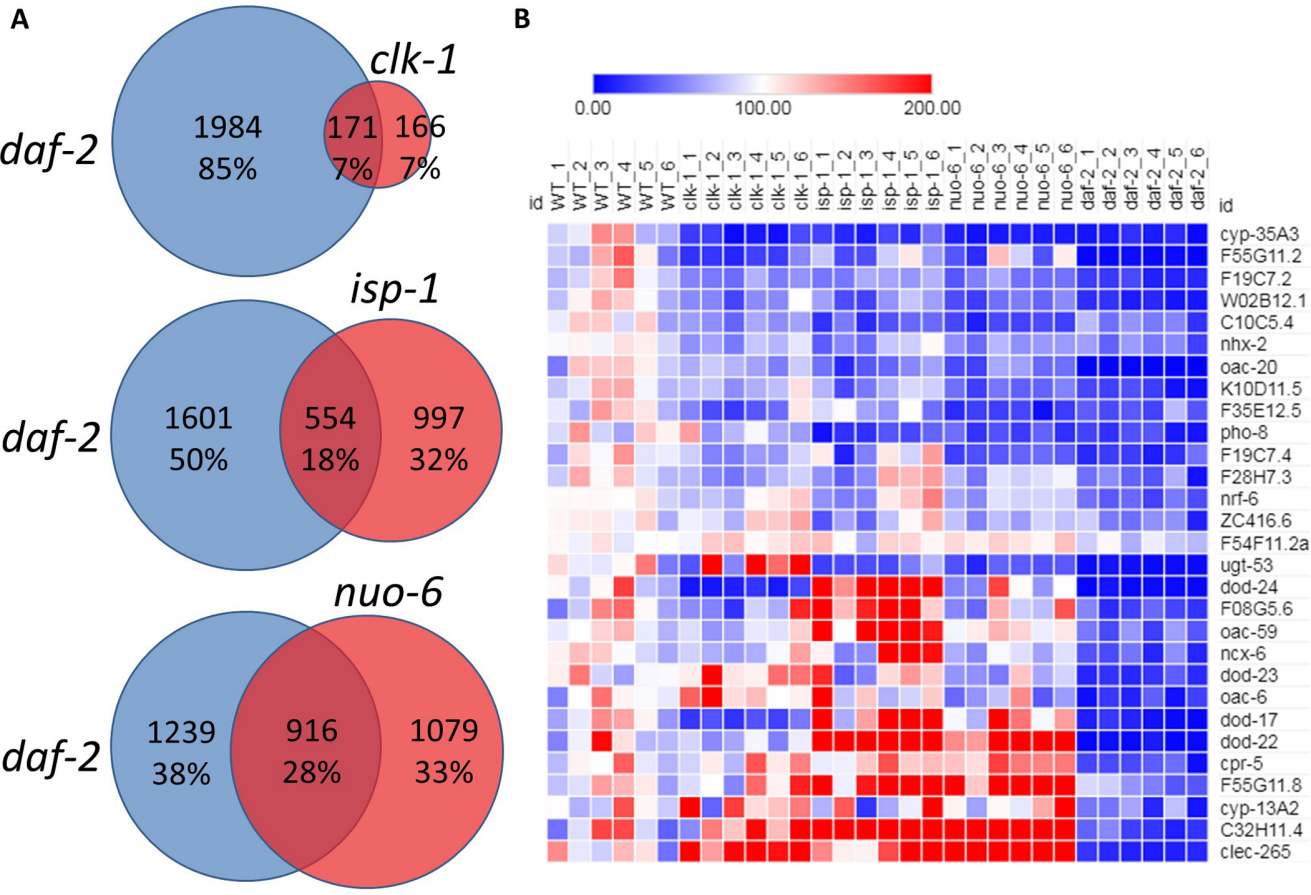
Overlap of downregulated genes between long-lived mitochondrial mutants and *daf-2* mutants. **A.** Of the genes that are downregulated in the long-lived mitochondrial mutant strains *clk-1*, *isp-1* and *nuo-6*, 51%, 36% and 42% respectively are also downregulated in *daf-2* mutants. **B.** A heat map showing the top DAF-16 responsive genes that are downregulated in *daf-2* worms genes (genes included are from the meta-analysis of DAF-16 target genes performed by Tepper *et al*., 2013 that were significantly downregulated in our *daf-2* RNAseq data). While many of these genes are also downregulated in the long-lived mitochondrial mutant strains, some are upregulated. mRNA for all strains was isolated from six biological replicates and sequenced individually.

Since the long lifespan of the insulin-IGF1 receptor mutant *daf-2* is thought to be mediated by DAF-16, we next sought to determine the extent to which gene expression changes present in the long-lived mitochondrial mutants overlapped with gene expression changes in *daf-2* worms. Accordingly, we performed RNA sequencing on *daf-2(e1370)* worms and compared the changes in gene expression to the long-lived mitochondrial mutants. Of the genes that were found to be upregulated in *clk-1*, *isp-1* and *nuo-6* worms, 46%, 50% and 57%, respectively, were also upregulated in *daf-2* mutants (**Fig 2A**), which represents a highly significant degree of overlap (**Table 1**). Of the genes that were found to be downregulated in *clk-1*, *isp-1* and *nuo-6* worms, 51%, 36% and 42%, respectively, were also downregulated in *daf-2* mutants (**Fig 3A**), which also represents a statistically significant degree of overlap (**Table 1**). Thus, there is considerable overlap between the transcriptional response in the long-lived mitochondrial mutants and decreasing insulin-IGF1 signaling through *daf-2* mutation.

**Table 1:** DAF-16 target gene enrichment in mitochondrial mutants. *clk-1*, *isp-1* and *nuo-6* mutants exhibit a highly significant overlap with *daf-2* mutants and enrichment of DAF-16 target genes. The number of genes exhibiting a significant change in expression for each strain are shown in brackets.

	Strain	Number of overlapping genes	Expected Number of overlapping genes	Significance
**Genes upregulated in *daf-2* mutants (2594)**	*clk-1* (838)	385	155	p < 0.000001
	*isp-1* (1511)	766	279	p < 0.000001
	*nuo-6* (3311)	1469	479	p < 0.000001
**Genes downregulated in *daf-2* mutants (2155)**	*clk-1* (337)	171	52	p < 0.000001
	*isp-1* (1551)	554	238	p < 0.000001
	*nuo-6* (1995)	906	306	p < 0.000001
**Top-ranked DAF-16 upregulated genes (39)**	*clk-1* (838)	10	2.3	p = 0.000071
	*isp-1* (1511)	24	4.2	p < 0.000001
	*nuo-6* (3311)	28	7.2	p < 0.000001
**Top-ranked DAF-16 downregulated genes (29)**	*clk-1* (337)	6	0.7	p = 0.000055
	*isp-1* (1551)	11	3.2	p = 0.000151
	*nuo-6* (1995)	8	4.4	p = 0.06

A previous study analyzed all gene expression studies that compared conditions with different levels of DAF-16 to identify DAF-16 up-regulated and DAF-16 down-regulated gene sets [27]. Accordingly, we compared our RNA sequencing results with the top-ranked DAF-16-modulated genes identified in the previous study. We focused on genes identified in the study by Tepper *et al*. that were significantly modulated in our *daf-2* RNAseq data. We found that the genes that are upregulated in *clk-1*, *isp-1* and *nuo-6* mutants are significantly enriched for the DAF-16 upregulated genes (**Fig 2; Table 1**) and that DAF-16-down-regulated genes are also down-regulated in the mitochondrial mutants (**Fig 3; Table 1**).

### Altered expression of DAF-16 target genes in long-lived mitochondrial mutants is mediated by DAF-16

**Fig 4:**
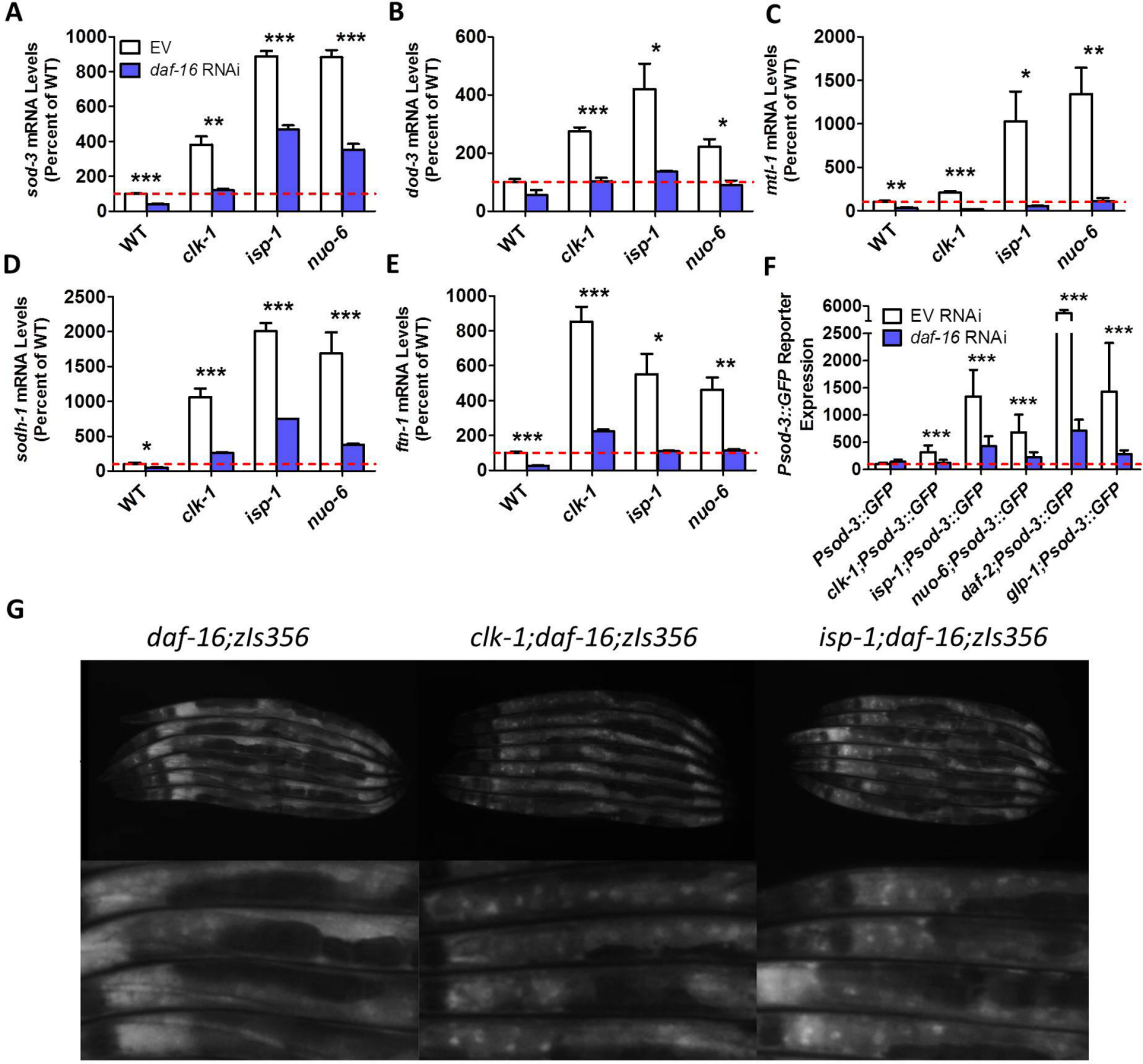
Increased expression of DAF-16 target genes in mitochondrial mutants is mediated by DAF-16. The expression of *daf-16* was knocked down by RNAi in wild-type, *clk-1*, *isp-1* and *nuo-6* worms, and the resulting effect on the expression of DAF-16 target genes was quantified by quantitative real time RT-PCR (qPCR). *daf-16* knockdown reduced expression of *sod-3* (**A**), *dod-3* (**B**), *mtl-1* (**C**), *sodh-1* (**D**), and *ftn-1* (**E**) in all strains. **F.** Similarly, activity of a *Psod-3*::*sod-3*:*GFP* reporter strain was reduced by *daf-16* RNAi. **G.** DAF-16 shows increased nuclear localization in *clk-1* and *isp-1* mitochondrial mutants. Combined these results indicate that DAF-16 is activated in the mitochondrial mutant strains leading to activation of DAF-16 target genes. Error bars indicate SEM. *p<0.05, **p<0.01, ***p<0.001.

To ensure that the upregulation of DAF-16 target genes in the long-lived mitochondrial mutants is being mediated by DAF-16, and not by other transcription factors that can activate the same genes, we knocked down *daf-16* expression in the mitochondrial mutant strains using RNAi, and compared gene expression to worms treated with an empty vector RNAi bacteria. We examined five DAF-16 target genes (*sod-3*, *dod-3*, *mtl-1*, *sodh-1* and *ftn-1*) and found that the increased expression of these genes in the long-lived mitochondrial mutants was significantly reduced (*sod-3*, *sodh-1*) or prevented (*dod-3*, *mtl-1*, *ftn-1*) by knocking down DAF-16 (**Fig 4A–4E**). This indicates that DAF-16 is required or partially required for the upregulation of the DAF-16 target genes in *clk-1*, *isp-1* and *nuo-6* worms. A DAF-16-dependent upregulation of *sod-3* in *isp-1* worms was also previously observed by others [5]. As with the quantitative real-time RT-PCR results, we found that the long-lived mitochondrial mutants exhibit increased fluorescence from a *Psod-3*::*GFP* reporter strain [34], which is diminished by *daf-16* RNAi (**Fig 4F**).

To further explore DAF-16 activation in the long-lived mitochondrial mutant strains, we examined the nuclear localization of DAF-16. When DAF-16 is activated it moves to the nucleus, and thus increased nuclear localization of DAF-16 is indicative of activation. Accordingly, we crossed *clk-1* and *isp-1* worms to a reporter strain expressing DAF-16 linked to GFP (*zIs356[Pdaf-16*::*daf-16*:*GFP]*)[24]. To ensure that we could visualize all of the DAF-16 protein present, we performed these experiments in a *daf-16* deletion mutant background, as has been done previously [35]. While control worms (*daf-16;zIs356*) exhibited diffuse cytoplasmic expression of DAF-16:GFP, both *clk-1* and *isp-1* worms showed increased nuclear localization of DAF-16:GFP compared to wild-type worms (**Fig 4G; S1 Fig**). Note that we could not test nuclear localization in *nuo-6* worms because we were unable to generate *nuo-6;daf-16;zIs356* worms due to the close proximity of *nuo-6* and *daf-16* on chromosome I. In addition, we did not explore the effect of *nuo-6* RNAi on DAF-16 nuclear localization since the *nuo-6* mutation and *nuo-6* RNAi increase lifespan by independent mechanisms and exhibit different changes in gene expression [6]. Nonetheless, our results indicate that DAF-16 is activated in long-lived mitochondrial mutant strains, leading to increased nuclear localization and altered expression of DAF-16 target genes.

### DAF-16/FOXO is required for the full longevity of long-lived mitochondrial mutants

**Fig 5:**
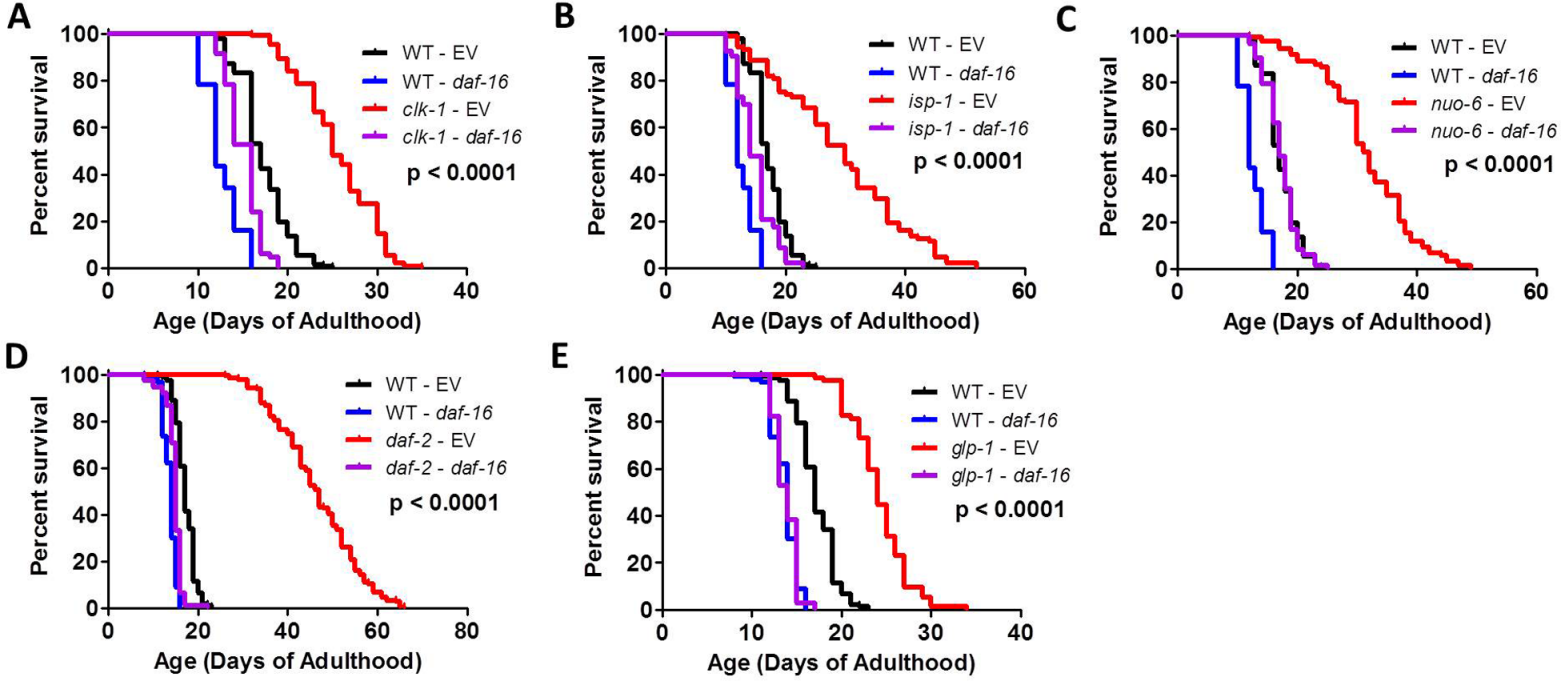
Lifespan of long-lived mitochondrial mutants is dependent on DAF-16/FOXO. The lifespan of the long-lived mitochondrial mutant strains *clk-1* (**A**), *isp-1* (**B**), and *nuo-6* (**C**) are all markedly reduced by knocking down *daf-16* by RNAi. Similarly, *daf-16* RNAi abolishes the increase in lifespan that results from decreasing insulin-IGF1 signaling through a mutation in *daf-2* (**D**), or germline ablation, through a mutation in *glp-1* (**E**). P-value indicates significance of difference between EV (red) and *daf-16* RNAi (purple) for the experimental strain. Data and N for the lifespan experiments are included in **S2 Table**.

The loss of DAF-16 has previously been shown to completely prevent the long lifespan of *daf-2* insulin-IGF1 receptor mutants [20] and *glp-1* mutants [36–38]. Since DAF-16 is activated in the long-lived mitochondrial mutants, and the changes in gene expression present in these mutants exhibit a high degree of overlap with those of *daf-2* worms, we examined the effect of knocking down *daf-16* levels by RNAi on the lifespan of *clk-1*, *isp-1* and *nuo-6* worms. In each case, we found that *daf-16* RNAi markedly decreased the lifespan of the long-lived mitochondrial mutant (**Fig 5A–5C**). Similarly, as had been previously observed [20,37], *daf-16* RNAi completely prevented mutations in either *daf-2* or *glp-1* from increasing lifespan (**Fig 5D and 5E**).

To rule out the possibility that *daf-16* RNAi was decreasing lifespan through off target effects, we also examined the effect of a *daf-16* deletion mutation on mitochondrial mutant longevity. We chose to use the mu86 allele as this mutation affects all transcripts of *daf-16*. We measured the lifespan of *clk-1;daf-16(mu86)*, *isp-1;daf-16(mu86)* and *daf-2;daf-16(mu86)* double mutants (we were unable to generate *nuo-6;daf-16(mu86)* worms due to the close proximity of *nuo-6* and *daf-16* on chromosome I).

**Fig 6:**
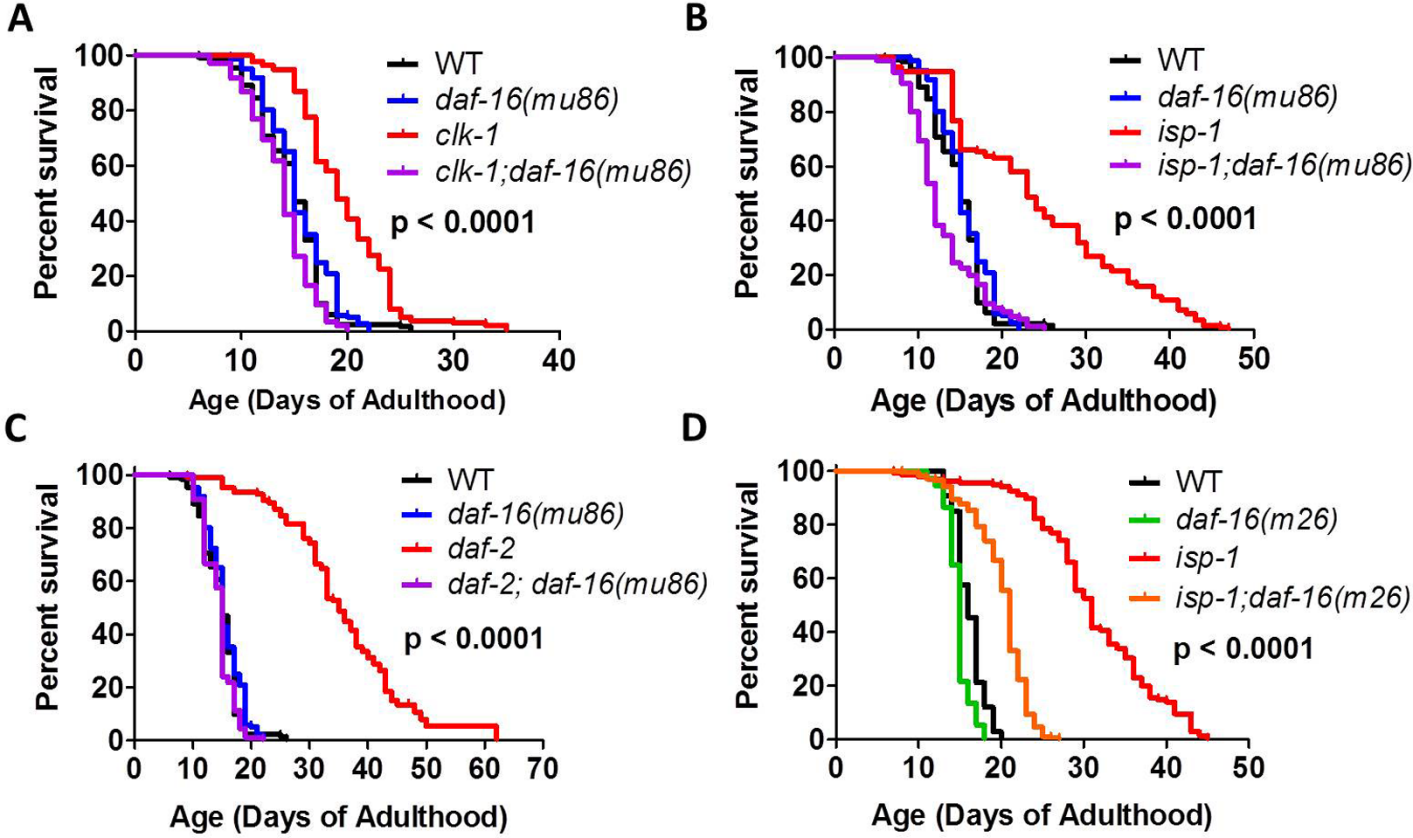
DAF-16 is required for the longevity of *clk-1*, *isp-1* and *daf-2* worms. The long lifespan of *clk-1* (**A**), *isp-1* (**B**) and *daf-2* (**C**) mutants is reduced to wild-type by the *daf-16(mu86)* deletion mutation. **D.** The *daf-16(m26)* point mutation also decreases *isp-1* lifespan but to a lesser extent than the *mu86* deletion mutation. P-value indicates significance of difference between control and *daf-16* mutation for the experimental strain. Data and N for the lifespan experiments are included in **S2 Table**.

As with *daf-16* RNAi, we found that a null mutation in *daf-16*(*mu86* allele) completely prevented the increase in lifespan observed in *clk-1*, *isp-1* and *daf-2* worms (**Fig 6**). Note that the effect of the *daf-16(mu86)* mutation on lifespan is dependent on the experimental conditions: at 20°C *daf-16(mu86)* worms exhibit a similar lifespan to wild-type worms (equal or mildly decreased), while at 25°C *daf-16(mu86)* worms show a marked decrease in longevity compared to wild-type worms (**S2 Fig**). We chose to perform these experiments at 20°C where the *daf-16(mu86)* mutation has either no effect on longevity (**Fig 6**) or causes a small reduction in lifespan (**S2 Fig**). In both cases, we found that deletion of *daf-16(mu86)* markedly reduced *clk-1* and *isp-1* lifespan (**Fig 6, S2 Fig**).

As previous work using a *daf-16* point mutation (m26) reported milder effects of *daf-16* mutation on mitochondrial mutant longevity [2,5], we sought to determine if the magnitude of lifespan decrease is related to the severity of the *daf-16* allele. Accordingly, we compared the lifespan of *isp-1* worms to *isp-1;daf-16(m26)* worms. As with the *daf-16(mu86)* allele, we found that the *daf-16(m26)* allele significantly decreased *isp-1* lifespan (**Fig 6**). The percentage decrease was 48% for the *daf-16(mu86)* allele and 36% for the *daf-16(m26)* allele, indicating that the milder allele has a lesser impact on *isp-1* longevity.

To further explore the role of DAF-16 in the lifespan of the long-lived mitochondrial mutants, we calculated the percentage increase in lifespan resulting from mutations in *clk-1*, *isp-1* and *nuo-6* under basal conditions and conditions in which *daf-16* expression is decreased. We found that the average increase in lifespan resulting from the *clk-1*, *isp-1*, and *nuo-6* mutation on EV RNAi was 48%, 72%, and 85% respectively. The average increase in lifespan resulting from the *clk-1*, *isp-1*, and *nuo-6* mutation on *daf-16* RNAi was 17%, 19%, and 34% respectively. In each case, the difference in magnitude of lifespan increase on EV RNAi is statistically different from *daf-16* RNAi (**S3 Fig**). Similarly, the maximum lifespan resulting from the *clk-1*, *isp-1*, and *nuo-6* mutations on EV RNAi was 45%, 99% and 90%, compared to 25%, 38% and 50% on *daf-16* RNAi. These results indicate that the ability of the mitochondrial mutations to increase lifespan is greatly diminished when *daf-16* is knocked down using RNAi. Similar results were obtained in analyzing the effect of the *daf-16* mutation (**S3 Fig**).

We next examined the effect of increasing DAF-16 levels on longevity in the mitochondrial mutant strains. To do this, we crossed *clk-1*, *isp-1* and *nuo-6* worms to *zIs356[Pdaf-16*::*daf-16*:*GFP]* worms. It should be noted that previous work has shown that the lifespan of *zIs356* worms is dependent on the concentration of FUdR in the media. While it was initially found that the lifespan of *zIs356* worms is similar to wild-type worms when FUdR is absent [24], subsequent studies showed that the lifespan of *zIs356* worms is increased compared to wild-type worms on plates containing FUdR [39,40]. We chose to complete our studies using conditions under which the *daf-16* transgene increases lifespan (100 μM FUdR). As previously reported, we found that when FUdR is present, *zIs356* worms exhibit increased lifespan (**S4A Fig**). Overexpression of DAF-16 also increased the lifespan of *clk-1*, *isp-1* and *nuo-6* worms, but not *daf-2* worms (**S4B–S4E Fig**). The ability of DAF-16 to increase lifespan was greatest in *clk-1* worms followed by *isp-1* worms, *nuo-6* worms and *daf-2* worms (**S4F Fig**). This pattern is inversely related to the magnitude of upregulation of DAF-16 target genes in the single mutant strains (**Fig 1**; *clk-1* < *isp-1* < *nuo-6* < *daf-2*). Combined these results suggest that there may be a threshold for DAF-16 activation with respect to longevity and that *daf-2* and *daf-2;zIs356* worms are at or above this threshold, while the individual mitochondrial mutants are all below the threshold such that lifespan continues to increase with further DAF-16 activation.

### Increasing levels of reactive oxygen species modulates expression of DAF-16 target genes

Having shown that DAF-16 is required for the longevity of the mitochondrial mutants as is true for *daf-2* worms, we next sought to determine the extent to which there is a common underlying mechanism. Previous work has demonstrated that the levels of reactive oxygen species (ROS) are increased in *clk-1*, *isp-1*, *nuo-6*, *daf-2* and *glp-1* mutants, and importantly that this increase in ROS is required for their longevity [14,41–44]. In addition, a number of experiments have shown that DAF-16 can be activated by ROS [24,35,45,46]. Accordingly, we hypothesized that one of the common underlying mechanisms of lifespan extension in all of these mutants would be the activation of DAF-16 by ROS.

**Fig 7:**
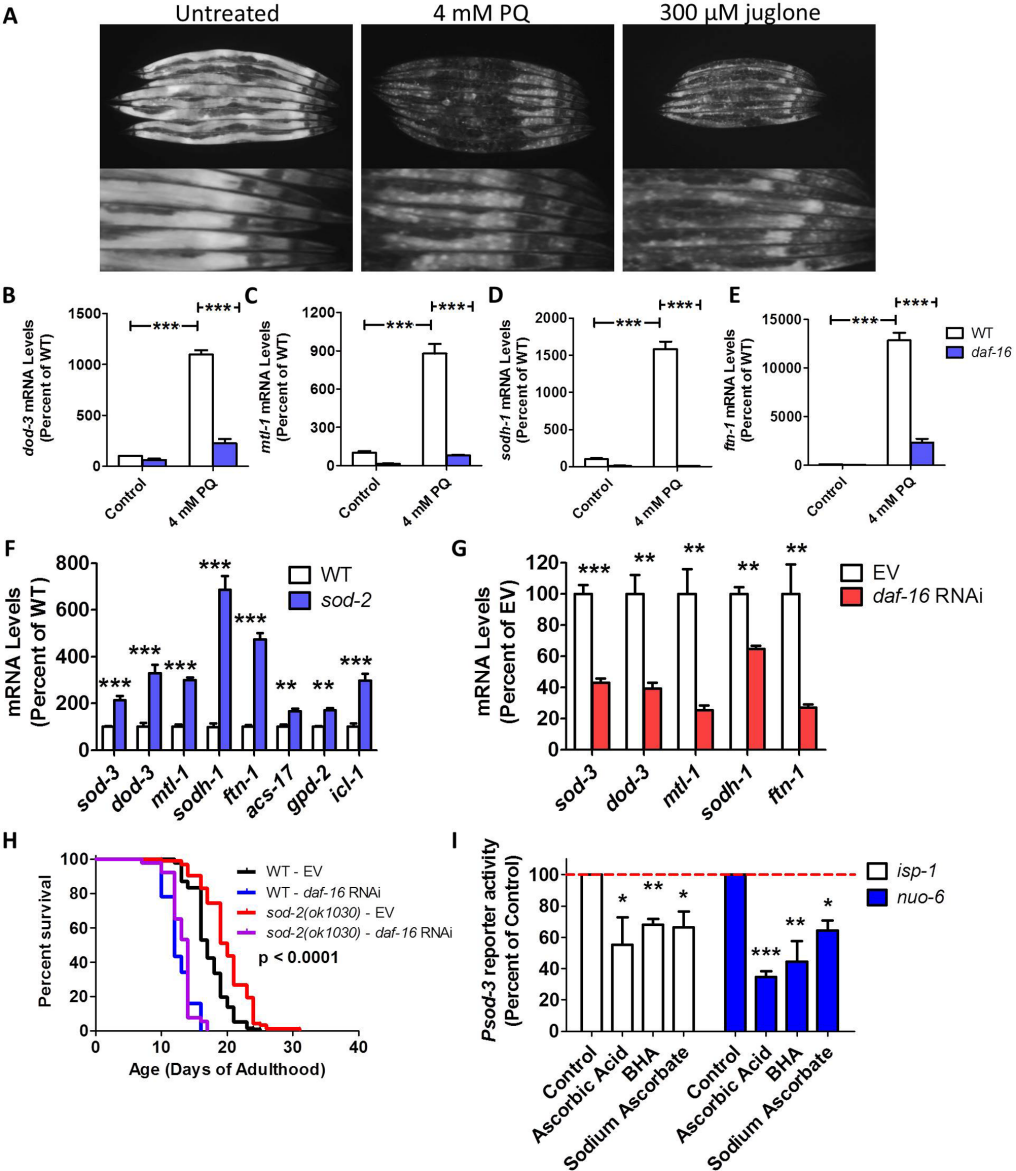
Reactive oxygen species cause nuclear localization of DAF-16 and activation of DAF-16 target genes. **A.** Increasing ROS levels through treatment with either 4 mM paraquat (PQ) or 300 μM juglone results in the nuclear localization of DAF-16. Young adult worms were treated with 300 μM juglone for 2 hours or 4 mM paraquat for 1 day. Age-matched untreated worms exhibited no nuclear localization of DAF-16:GFP (controls for 4 mM paraquat treated worms are shown). In wild-type worms, treatment with 4mM paraquat results in upregulation of DAF-16 target genes *dod-3* (**B**), *mtl-1* (**C**), *sodh-1* (**D**), and *ftn-1* (**E**). In contrast, this increase in gene expression resulting from elevated ROS is markedly reduced in the *daf-16(mu86)* deletion mutant (**B-E**)**. F.** As in the long-lived mitochondrial mutants and worms treated with 4 mM paraquat, multiple DAF-16 target genes are upregulated in *sod-2* deletion mutants. **G.** The upregulation of the DAF-16 target genes in *sod-2* worms is dependent on DAF-16 as knocking down *daf-16* mRNA using RNAi decreased the upregulation of DAF-16 target genes in *sod-2* worms. **H.** *daf-16* RNAi decreases the long lifespan of *sod-2* worms, indicating that DAF-16 is required for their longevity. P-value indicates significance of difference between EV (red) and *daf-16* RNAi (purple) for *sod-2* worms. **I.** Activation of the DAF-16 target gene *sod-3* in *isp-1* and *nuo-6* worms was reduced by treatment with antioxidants: 10 mM ascorbic acid, 25 μM butylated hydroxyanisole (BHA) or 10 mM sodium ascorbate. Error bars indicate SEM. *p<0.05, **p<0.01, ***p<0.001. Data and N for the lifespan experiments are included in **S2 Table**.

To test this hypothesis, we examined the effect of increasing ROS on the nuclear localization of DAF-16 and expression of DAF-16 target genes. We treated worms with two different compounds that increase intracellular levels of ROS: 4 mM paraquat or 300 μM juglone. As had been previously observed [24], we found that increasing ROS resulted in nuclear localization of DAF-16:GFP, similar to what we had observed in *clk-1* and *isp-1* mutants (**Fig 7A, S1 Fig**). Increasing the levels of ROS through treatment with 4 mM paraquat also resulted in the upregulation of DAF-16 target genes, including *dod-3*, *mtl-1*, *sodh-1* and *ftn-1* (**Fig 7B–7E**). Importantly, the upregulation of these genes was prevented by the *daf-16(mu86)* mutation (**Fig 7B–7E**). Note that we and others have previously shown that treating worms with either paraquat or juglone can extend longevity [14,41,47,48].

To further explore the role of elevated ROS, we examined gene expression in *sod-2* mutants, since these worms have a decreased ability to specifically detoxify mitochondrial ROS and, similar to the mitochondrial mutants, have increased lifespan [8]. As with the long-lived mitochondrial mutants, we found that many DAF-16 target genes are upregulated in *sod-2* worms (**Fig 7F**; see **Fig 1C–1J** to compare to results from mitochondrial mutants), and that the upregulation of these genes is dependent on DAF-16 (**Fig 7G**; see **Fig 3A–3E** to compared to results from mitochondrial mutants).

Next, we compared RNAseq results between *sod-2* and *daf-2* worms. Among the genes that are upregulated in *sod-2* mutants, 36% are also upregulated in *daf-2* worms (**S5 Fig**). Among the genes that are downregulated in *sod-2* worms, 24% are also downregulated in *daf-2* worms (**S5 Fig**). Thus, as with the mitochondrial mutants, there is considerable overlap in the transcriptional changes present in *sod-2* and *daf-2* worms. Since *sod-2* can be transcriptionally regulated by DAF-16, it is possible that the overlap in gene expression between *sod-2* and *daf-2* worms could results from being in the same pathway (*daf-2* → *daf-16* → *sod-2* → *daf-16* target genes), but, given the function of *sod-2*, we believe it is more likely that the elevated ROS in *sod-2* mutants causes DAF-16 activation. Finally, we examined the effect of *daf-16* RNAi on *sod-2* lifespan and found that the long life of *sod-2* worms is completely abolished by *daf-16* RNAi (**Fig 7H**). Combined, our data suggests that elevated ROS can cause the nuclear localization of DAF-16, activation of DAF-16 target genes, and a DAF-16-dependent increase in lifespan.

### Decreasing ROS levels reduces expression of DAF-16 target gene in long-lived mitochondrial mutants

Having shown that increasing ROS levels can activate DAF-16 target genes, we next sought to determine whether elevated levels of ROS contribute to modulation of DAF-16 target genes in the long-lived mitochondrial mutants. First, we sought to confirm that ROS levels are increased in the mitochondrial mutant strains, and determine whether this increase is dependent on DAF-16. For this purpose, we stained wild-type, *daf-16*, *clk-1*, *clk-1;daf-16*, *isp-1* and *isp-1;daf-16* worms with the ROS-sensitive dye dihydroethidium (DHE). As we and others have previously observed [14,49], DHE fluorescence is significantly increased in *clk-1* and *isp-1* worms (**S6 Fig**). The increase in ROS levels in these mutants is independent of DAF-16, as *clk-1;daf-16* and *isp-1;daf-16* mutants both show elevated levels of ROS (**S6 Fig**). This indicates that DAF-16 activation does not contribute to the elevated ROS levels in these long-lived mitochondrial mutants.

Having shown that ROS levels are increased in the long-lived mitochondrial mutants, we next sought to determine whether these ROS activate DAF-16. If elevated ROS in the long-lived mitochondrial mutants are causing activation of DAF-16 leading to upregulation of DAF-16 target genes, then decreasing ROS levels through treatment with an antioxidant should prevent the upregulation of DAF-16 target genes. To test this we used the *Psod-3*::*GFP* reporter strain. We treated *isp-1;Psod-3*::*GFP* and *nuo-6;Psod-3*::*GFP* worms with three different antioxidants that have previously been shown to decrease levels of ROS in worms: ascorbic acid, butylated hydroxyanisole and sodium ascorbate [42,43]. In each case, we found that the antioxidant treatment decreased activation of the *Psod-3*::*GFP* reporter (**Fig 7I**), suggesting that reporter was being activated by elevated ROS. We previously showed that this same antioxidant treatment decreases the lifespan of *isp-1* worms [47], *clk-1* worms [50], and *sod-2* worms [51]. While these results indicate that decreasing ROS levels with antioxidants, reduces the expression of the DAF-16 target gene *sod-3*, they do not exclude the possibility that other transcription factors, such as SKN-1, contribute to the ROS-mediated upregulation of *sod-3* and other DAF-16 target genes.

### DAF-16 deubiquitylase MATH-33 is required for the longevity of long-lived mitochondrial mutants

**Fig 8:**
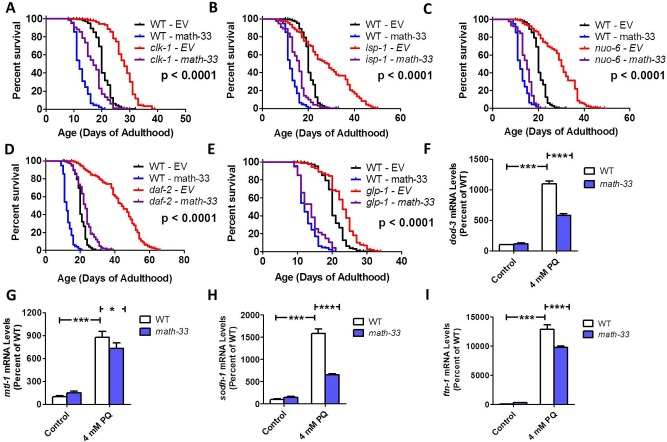
Deubiqutitylase MATH-33/USP7 is required for lifespan of long-lived mutants and upregulation of DAF-16 target genes by reactive oxygen species. Decreasing the expression of *math-33* by RNAi reduced the lifespan of the long-lived mitochondrial mutants *clk-1* (**A**), *isp-1* (**B**), and *nuo-6* (**C**). Similarly, knockdown of *math-33* reduced the lifespan of both *daf-2* mutants (**D**), and *glp-1* mutants (**E**). This indicates that MATH-33 is required for the longevity of the long-lived mitochondrial mutants. A mutation in *math-33* decreases the upregulation of the DAF-16 target genes in response to reactive oxygen species (4 mM paraquat (PQ) treatment) but not under basal conditions: *dod-3* (**F**), *mtl-1* (**G**), *sodh-1* (**H**), and *ftn-1* (**I**). Error bars indicate SEM. *p<0.05, **p<0.01, ***p<0.001. P-value indicates significance of difference between EV (red) and *math-33* RNAi (purple) for the experimental strain. Data and N for the lifespan experiments are included in **S2 Table**.

It was recently shown that the deubiquitylase MATH-33/USP7 stabilizes DAF-16 during decreased insulin-IGF1 signaling by reducing DAF-16 ubiquitylation thereby preventing degradation by the proteasome [52]. As with mutations in *daf-16*, a loss of function mutation in *math-33* was found to completely abolish the increased lifespan of *daf-2* mutants. To determine if MATH-33 is also required for the longevity of the long-lived mitochondrial mutants, we knocked down *math-33* expression using RNAi. We found that *math-33* RNAi markedly reduced the lifespan of *clk-1*, *isp-1* and *nuo-6* worms (**Fig 8A–8C**). In accordance with previous work [52], *math-33* RNAi shortened the lifespan of *daf-2* worms (**Fig 8D**). Finally, we tested the role of *math-33* in *glp-1* germline ablation mutants and found that knocking down *math-33* completely prevented their increase in lifespan (**Fig 8E**). To confirm the results of the RNAi experiments, we generated *isp-1;math-33* and *nuo-6;math-33* double mutants (note that we were unable to generate *clk-1;math-33* double mutants possibly due to lethality). As with RNAi knockdown, deletion of *math-33* also reduced the long life of both *isp-1* and *nuo-6* mutants (**S7 Fig**).

To test the extent to which *math-33* is required for the upregulation of DAF-16 target genes in response to elevated ROS, we treated wild-type worms and *math-33* deletion mutants with 4 mM paraquat and examined the expression of four DAF-16 target genes using quantitative real-time RT-PCR. We found that in each case, the ability of ROS to activate DAF-16 targets was diminished in *math-33* mutants (**Fig 8F and 8I**).

### DAF-16-target-regulating transcription factor PQM-1 is required for the longevity of long-lived mitochondrial mutants

**Fig 9:**
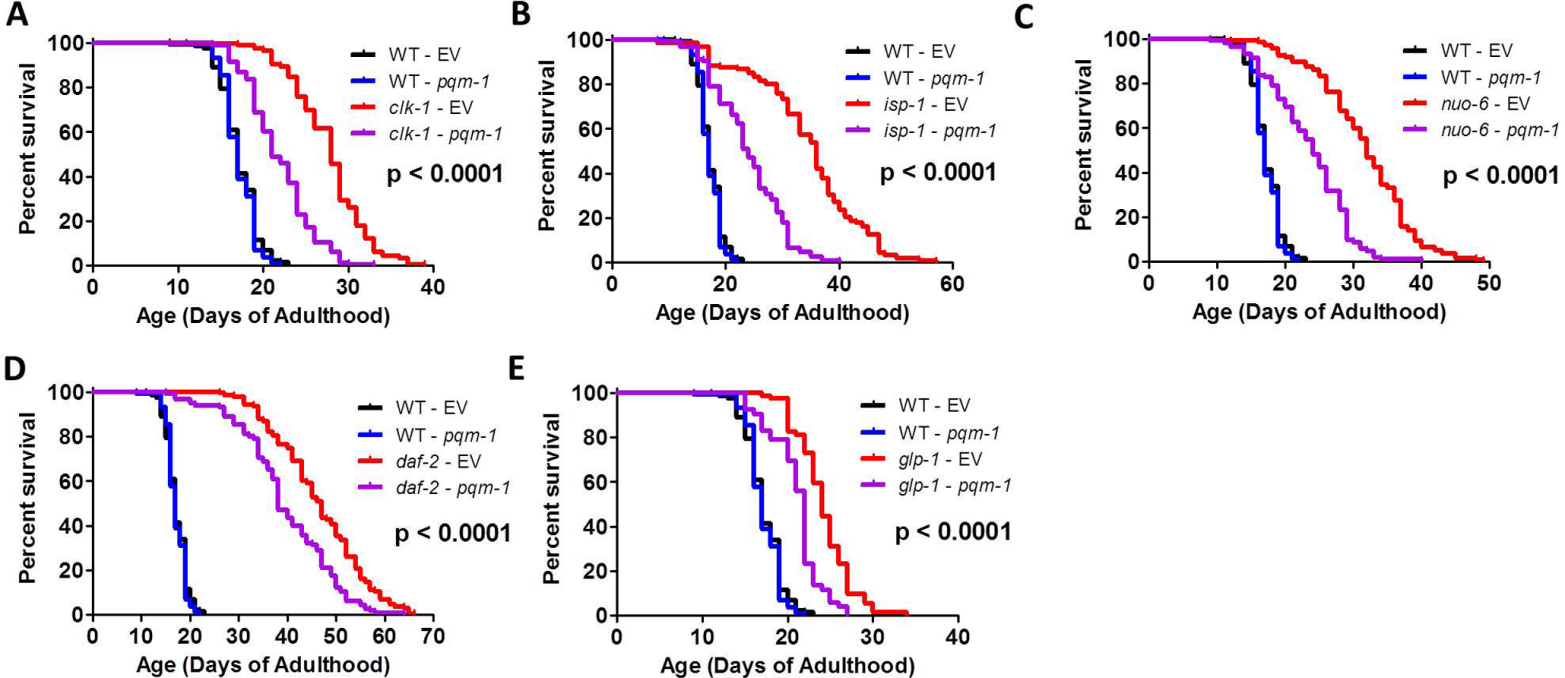
The transcription factor PQM-1 is required for longevity of long-lived mitochondrial mutants. Decreasing *pqm-1* expression by RNAi decreased the lifespan of *clk-1* (**A**), *isp-1* (**B**), and *nuo-6* (**C**) worms. Similarly, knockdown of *pqm-1* partially reduced the lifespan of *daf-2* (**D**), and *glp-1* (**E**) mutants. P-value indicates significance of difference between EV (red) and *pqm-1* RNAi (purple) for the experimental strain. Data and N for the lifespan experiments are included in **S2 Table**.

The activation of DAF-16 target genes, especially those that are downregulated in response to decreased insulin-IGF1 signaling, have been shown to be co-regulated by the transcription factor PQM-1 [27]. PQM-1 appears to act in conjunction with DAF-16 to promote growth and development, or repair and maintenance depending on the environmental conditions. Accordingly, we tested the role of PQM-1 in the lifespan of the long-lived mitochondrial mutants. We found that knocking down *pqm-1* expression using RNAi partially reduced the lifespan of *clk-1*, *isp-1* and *nuo-6* mutants (**Fig 9A–9C**). Similarly, *pqm-1* RNAi also reduced the lifespan of *daf-2*, as previously noted [27], and *glp-1* mutants (**Fig 9D–9F**).

### Proteins that modify DAF-16/FOXO in response to oxidative stress are required for the longevity of long-lived mitochondrial mutants

Multiple proteins have been shown to interact with DAF-16 under conditions of elevated ROS to promote DAF-16 activation, nuclear localization of DAF-16, and changes in the expression of DAF-16 target genes. The protein kinase MST1 has been shown to phosphorylate FOXO in response to treatment with ROS, thereby disrupting its interaction with 14-3-3 proteins and promoting its nuclear localization [46]. Overexpression of *cst-1*, the *C*. *elegans* homolog of MST1, increases lifespan in a *daf-16-*dependent manner, while *cst-1* RNAi reduces the lifespan of *daf-2* worms [46]. Similarly, β-catenin has been shown to bind to FOXO in order to increase FOXO transcriptional activity in response to ROS. Loss of *bar-1*, the *C*. *elegans* homolog of β -catenin, results in decreased lifespan, and decreased upregulation of the DAF-16 target gene *sod-3* in response to paraquat [45]. Finally, the nuclear import receptor transportin-1 (TNPO1) has been shown to form a disulfide complex with FOXO4 in response to ROS leading to nuclear localization of FOXO4 [35]. This mechanism is conserved in *C*. *elegans* as knockdown of the *C*. *elegans* homolog of transportin-1 *imb-2* prevents the nuclear localization of DAF-16:GFP in response to paraquat-induced ROS [35].

**Fig 10:**
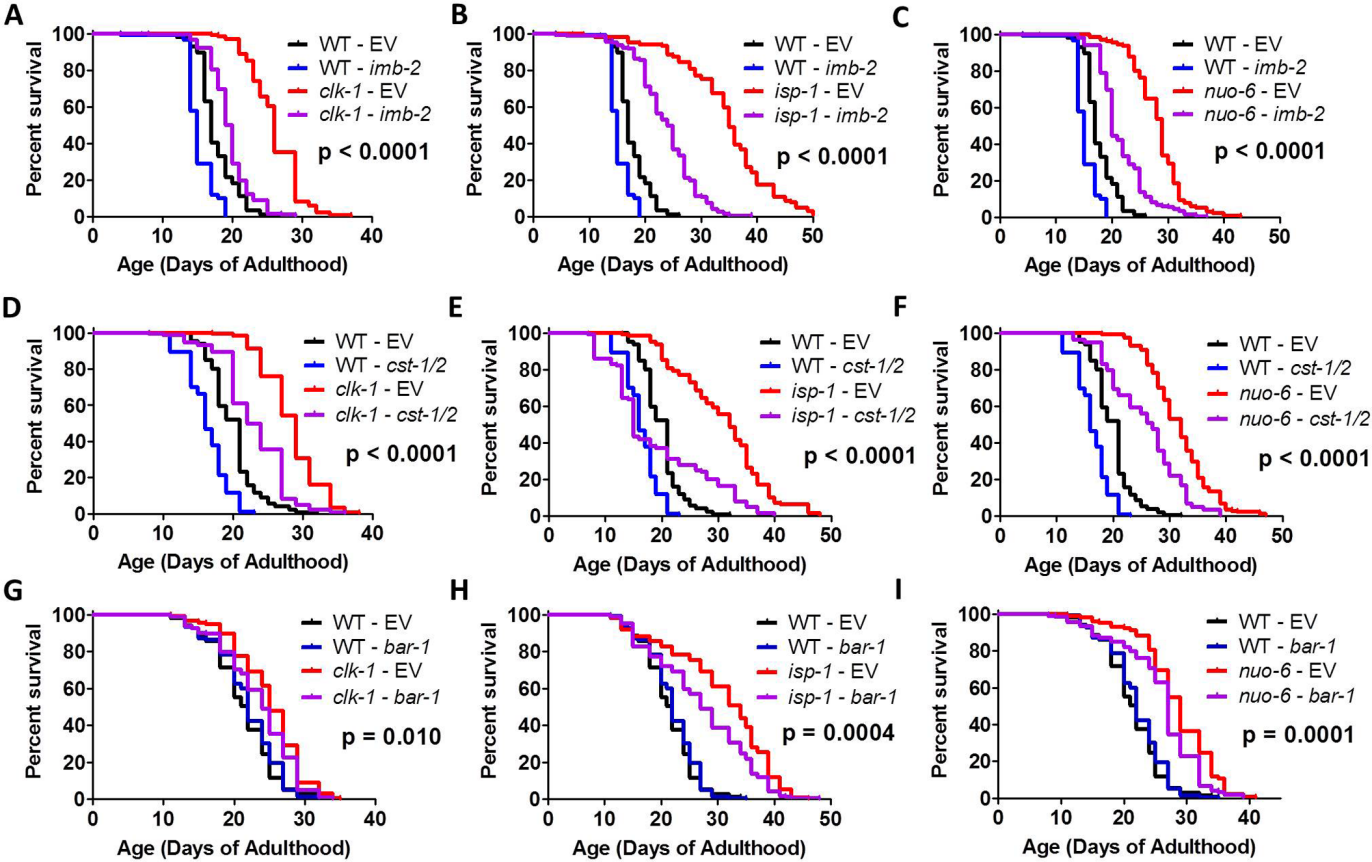
Lifespan of long-lived mitochondrial mutants is dependent on DAF-16 interacting proteins IMB-2, CST-1/CST-2 and BAR-1. Decreasing the expression of the transportin-1 homolog *imb-2* by RNAi decreases the lifespan of *clk-1* (**A**), *isp-1* (**B**)and *nuo-6* (**C**) mutants. Knockdown of the protein kinase *cst-1/cst-2* using RNAi also decreases the lifespan of *clk-1* (**D**), *isp-1* (**E**)and *nuo-6* (**F**) mutants. Decreasing expression of the B-catenin homolog *bar-1* results in a mild, but significant, decrease in *clk-1* (**G**), *isp-1* (**H**), and *nuo-6* (**I**) lifespan. A single RNAi clone was used to target *cst-1* and *cst-2*, which is indicated as *cst-1/2*. RNAi for *imb-2* was initiated at the egg stage of the experimental generation. RNAi for *cst-1/2* was initiated at the L4 stage of the parental generation. RNAi for *bar-1* was initiated at the L4 stage of the experimental generation since longer treatment with *bar-1* RNAi resulted in protruding vulva and externalization of internal organs. P-value indicates significance of difference between EV (red) and *imb-2/cst-1/2/bar-1* RNAi (purple) for the experimental strain. Data and N for the lifespan experiments are included in **S2 Table**.

Because CST-1/CST-2, BAR-1 and IMB-2 act with DAF-16 to upregulate DAF-16 target genes in response to ROS, we hypothesized that these genes might be important for the longevity of the long-lived mitochondrial mutants. Accordingly, we tested the effect of knocking down these three DAF-16 interacting proteins on the lifespan of *clk-1*, *isp-1* and *nuo-6* worms. We found that knocking down *imb-2* or *cst-1/cst-2* substantially decreased the lifespan of all three mitochondrial mutants (**Fig 10A–10F**). RNAi against *imb-2* or *cst-1/cst-2* also decreased the lifespan of *daf-2* and *glp-1* mutants (**S8 Fig**). However, it should be noted that the magnitude of the effect on *daf-2* and *glp-1* lifespan was less than in the mitochondrial mutants. Finally, we found that knockdown of *bar-1* resulted in a small but significant reduction in *clk-1*, *isp-1* and *nuo-6* lifespan (**Fig 10G–10I**), but did not affect the longevity of *daf-2* mutants (**S8 Fig**).

While the effect of knocking down *bar-1* on mitochondrial mutant longevity was milder than knockdown of *imb-2* or *cst-1/2*, it should be noted that the RNAi paradigm used was different. Because knockdown of *bar-1* throughout development causes protruding vulva and externalization of internal organs even in wild-type worms, we began RNAi knockdown at the L4 stage, whereas *imb-2* and *cst-1/2* RNAi was begun before development. Nonetheless, the fact that knocking down any of these genes significantly decreases lifespan in all three mitochondrial mutants demonstrates that proteins that facilitate the activation and nuclear localization of DAF-16 in response to ROS are required for the full lifespan of the long-lived mitochondrial mutants.

## Discussion

### Reactive oxygen species as a common mediator of longevity

The Free Radical Theory of Aging proposes that ROS produced by normal metabolism are one of the primary causes of aging. While it is clear that high levels of ROS are toxic and can decrease lifespan, it has been shown that mildly increasing ROS can be beneficial. Deletion of *sod-2*, the primary mitochondrial *sod* gene, causes increased lifespan [8], which is dependent on elevated ROS, since treatment with antioxidants reduces *sod-2* lifespan [41,51]. The ability of ROS to increase lifespan depends on the location of ROS within the cell: while mitochondrial superoxide increases lifespan, cytoplasmic ROS decreases it [50,53]. Similarly, it has been shown that treating worms with ROS-generating compounds, either paraquat or juglone, both result in increased lifespan [14,41,47,48]. In addition, there have been multiple examples of genetic mutations or interventions that increase lifespan in which the increase in lifespan has been shown to be at least partially caused by elevated ROS [54–59], including the long-lived mitochondrial mutant strains studied here.

*clk-1* worms have been shown to have increased levels of ROS [14,41,60,61] and this increase in ROS is required for their longevity [50]. Similarly, *isp-1* worms exhibit increased levels of mitochondrial ROS [14,41,62], and decreasing this ROS through treatment with an antioxidant limits their longevity [41,47]. Finally, *nuo-6* worms have elevated levels of mitochondrial superoxide [41], which is also required for their long lifespan [41].

While the mechanism underlying the longevity of *daf-2* mutants was thought to be through a direct effect of the *daf-2* mutation decreasing insulin-IGF1 signaling and cytoplasmic retention of DAF-16, it was demonstrated that elevated levels of ROS also contribute to the long lifespan of *daf-2* worms [42]. Combined, this suggests that DAF-16 is activated by two routes in *daf-2* mutants: decreased insulin-IGF1 signaling and increased ROS. Similarly, the mechanism underlying lifespan extension in *glp-1* germline ablation mutants was thought to be mediated by DAF-16 as deletion of *daf-16* completely prevents the increased lifespan of *glp-1* mutants [36–38,63]. More recent evidence suggests that elevated ROS also contribute to the longevity of *glp-1* mutants. These worms were shown to have increased levels of mitochondrial ROS, and quenching ROS through treatment with antioxidants reduced their lifespan [43]. Combined these results indicate that elevated ROS contribute to the longevity of mutants in multiple different pathways of lifespan extension.

It is important to note that the ability of ROS to increase lifespan is conserved across species. In yeast, increasing mitochondrial ROS, directly through treatment with menadione, or indirectly with rapamycin, causes increased chronological lifespan [64,65]. In flies, expression of the NADH dehydrogenase NDI1 [66], or knocking down the expression of complex I subunit ND75 [10], causes an increase in ROS that leads to extended longevity. Similar to *clk-1* mutant worms, which have increased ROS that contributes to their longevity [1,2], *Mclk1+/-* mice also exhibit increased ROS and long life [13]. Finally, D-glucosamine was shown to increase lifespan in both worms and in mice as a result of increased levels of ROS [58].

In addition to acting through DAF-16, it is likely that mild elevation of ROS also increase lifespan through NRF2/SKN-1. SKN-1 is activated in response to oxidative stress, and targets of SKN-1 have been shown to be activated in *clk-1*, *isp-1*, *nuo-6*, *daf-2* and *glp-1* mutants, as well as by treatment with paraquat [28,43,49,50,67]. SKN-1 has been shown to be required for the increased longevity observed in *isp-1*, *daf-2* and *glp-1* mutants [43,68]. Interestingly, there are multiple examples of lifespan extending pathways in which both DAF-16 and SKN-1 are involved [68–70]. Thus, an important area for future research will be to define how DAF-16 and SKN-1 interact in response to elevated ROS to promote longevity.

### DAF-16/FOXO as a common mediator of longevity

As the mechanisms by which elevated ROS extend lifespan have not been fully elucidated, we sought to gain insight into these mechanisms by examining overlapping changes in gene expression in three long-lived mitochondrial mutants. We found that there is a highly significant enrichment of DAF-16 target genes among the genes that are modulated in *clk-1*, *isp-1* and *nuo-6* worms and that *daf-16* is required for their longevity. On the surface, our results are surprising as they contradict a well-established belief in the aging research field that mild-impairment of mitochondrial function increases lifespan in a DAF-16-independent manner. This conclusion was reached by multiple different studies [2,5,7,19,32,33]. In these previous studies, it was shown that mild impairment of mitochondrial function by genetic mutation or RNAi increased the lifespan of both wild-type and *daf-16* mutants. From these data, it was concluded that the lifespan increase caused by mild impairment of mitochondrial function is DAF-16 independent, and this idea has persisted in the field [71]. However, the data from these earlier papers also clearly show that a mutation in *daf-16* decreases the long lifespan induced by RNAi or genetic mutations affecting mitochondrial genes, which is entirely consistent with our data, and indicates that *daf-16* is required for the full increase in longevity caused by mild impairment of mitochondrial function.

The interpretation of this data is complicated by the fact that *daf-16* RNAi or a mutation in *daf-16* can decrease lifespan in wild-type worms. As a result, more evidence is needed to either support or rule out a role for DAF-16 in the longevity resulting from mild impairment of mitochondrial function. Our new data showing (1) increased nuclear localization of DAF-16 in mitochondrial mutant strains, (2) upregulation of DAF-16 target genes in mitochondrial mutant strains, (3) a significant overlap in gene expression changes in the mitochondrial mutants and *daf-2* worms, and (4) that multiple DAF-16 interacting proteins are also required for the long life of the mitochondrial mutant strains, provides strong support for the interpretation of the data in which the lifespan increase resulting from mild impairment of mitochondrial function is DAF-16 dependent, but that other DAF-16-independent pathways also contribute to their longevity.

In comparing our results to previous studies it should be noted that these previous studies primarily involved RNAi against different mitochondrial genes than those studied here using genetic mutations. In addition to the fact that different genes were targeted, it is also important to note that the mechanism of lifespan extension has been shown to be different between genetic mutations affecting the mitochondria and RNAi against genes encoding mitochondrial proteins [6]. Thus, additional studies would need to be performed to make firm conclusions about the DAF-16 dependency resulting from RNAi targeting mitochondrial proteins.

Similarly, while two previous studies have shown that the lifespan increase resulting from treatment with low concentrations of paraquat is reduced by a *daf-16* mutation [14,41], further studies were not performed to determine the role of DAF-16 in increasing lifespan. Our current results show that paraquat treatment causes the nuclear localization of DAF-16, and a *daf-16-*dependent upregulation of DAF-16 target genes. Similarly, we show that *sod-2* deletion mutants exhibit a *daf-16-*dependent upregulation of DAF-16 target genes and that deletion of *daf-16* abolishes the lifespan increase in *sod-2* worms. Combined, these results indicate that DAF-16 is required for the enhanced longevity that results from elevated ROS.

In previous studies involving genetic mitochondrial mutants, it was found that a mutation in *daf-16(m26)* only mildly reduced the maximum lifespan of *clk-1(e2519)* worms but did not reduce mean lifespan [2] and that a *daf-16(m26)* mutation reduced the lifespan of *isp-1* worms [5], but to a lesser extent than we observed in our study. To determine whether the magnitude of lifespan decrease caused by *daf-16(mu86)* mutation in our study and this previous study stems from the specific allele of *daf-16* that was studied, we examined the effect of the two different *daf-16* mutations, *mu86* and *m26*, on the lifespan of *isp-1* worms. The *mu86* allele that we utilized is a null allele resulting from a deletion that affects all of the *daf-16* isoforms, while the *m26* allele is a point mutation (G to A transition) that affects a splicing site. This mutation should not affect DAF-16 isoforms a, e and g, since these isoforms do not contain the exon bearing the *m26* mutation. Importantly, *daf-16* isoform a has been shown to regulate longevity [30,72]. In addition, two abnormally spliced species were detected in *daf-16(m26)* mutants [22] and this mutant fails to completely suppress dauer formation in *daf-2* mutants [73]. While both *daf-16* alleles decreased the lifespan of *isp-1* mutants, the magnitude of this decrease was greater for the *mu86* allele than the *m26* allele, indicating that the impact on longevity is related to the severity of the *daf-16* mutation.

### Conclusions

Overall, this work indicates that there is considerable overlap between different pathways of lifespan extension, which were previously thought to be distinct. Just as ROS-mediated signaling now appears to play an important role in the lifespan extension resulting from mild impairment of mitochondrial function [41,50], decreased insulin-IGF1 signaling [42], germline ablation [43], dietary restriction [54] and transient hypoxia [74], our work demonstrates that DAF-16 is required for the long lifespan resulting from mild impairment of mitochondrial function, and, combined with previous work, indicates that DAF-16 is a common downstream mediator of longevity in multiple pathways of lifespan extension.

## Materials and methods

### Strains

Strains were maintained at 20°C on nematode growth media (NGM) plates seeded with OP50 bacteria. The following strains were used in these experiments. N2(WT), *clk-1(qm30)*, *isp-1(qm150)*, *nuo-6(qm200)*, *sod-2(ok1030)*, *daf-2(e1370)*, *glp-1(e2141)*, CF1553 *muIs84[Psod-3*::*GFP*,*rol-6]*, *daf-16(mu86)*, *math-33(tm6724)*, TJ356 *zIs356[Pdaf-16*:*daf-16a/b*::*GFP+rol-6(su1006)]*, DR26 *daf-16(m26)*, MQ1050 *isp-1(qm150);daf-16(m26)*. The following mutant strains were generated as previously described [75]:

JVR456 *isp-1(qm150); math-33(tm6724)*

JVR457 *nuo-6(qm200); math-33(tm6724)*

JVR176 *isp-1(qm150); muIs84[Psod-3*::*GFP+rol-6]*

JVR181 *clk-1(qm30); muIs84[Psod-3*::*GFP+rol-6]*

JVR307 *daf-2 (e1370); muIs84[Psod-3*::*GFP+rol-6]*

JVR308 *glp-1 (e2141); muIs84[Psod-3*::*GFP+rol-6]*

JVR317 *nuo-6(qm200); muIs84[Psod-3*::*GFP+rol-6]*

JVR303 *sod-2(ok1030); muIs84[Psod-3*::*GFP+rol-6]*

JVR297 *clk-1 (qm30); zIs356[Pdaf-16*:*daf-16a/b*::*GFP+rol-6(su1006)]*

JVR299 *nuo-6(qm200); zIs356[Pdaf-16*:*daf-16a/b*::*GFP+rol-6(su1006)]*

JVR302 *isp-1(qm150); zIs356[Pdaf-16*:*daf-16a/b*::*GFP+rol-6(su1006)]*

JVR304 *daf-2(e1370); zIs356[Pdaf-16*:*daf-16a/b*::*GFP+rol-6(su1006)]*

JVR316 *clk-1(qm30); daf-16(mu86)*

JVR327 *daf-2 (e1370); daf-16(mu86)*

JVR380 *isp-1(qm150);daf-16(mu86)*

JVR333 *daf-16(mu86) I; zIs356[Pdaf-16*:*daf-16a/b*::*GFP+rol-6(su1006)]*

JVR334 *clk-1(qm30); daf-16(mu86);zIs356[Pdaf-16*:*daf-16a/b*::*GFP+rol-6(su1006)]*

JVR335 *isp-1(qm150);daf-16(mu86);zIs356[Pdaf-16*:*daf-16a/b*::*GFP+rol-6(su1006)]*

We were unable to generate *nuo-6(qm200);daf-16(mu86)* or *nuo-6(qm200);daf-16(mu86);zIs356[Pdaf-16*:*daf-16a/b*::*GFP+rol-6(su1006)]* worms because of the close proximity of *nuo-6* and *daf-16* on chromosome I. We were also unable to generate *clk-1(qm30);math-33(tm6724)* double mutants. *clk-1;math-33+/-* worms repeatedly failed to produce any *clk-1;math-33* homozygous offspring suggesting that this combination might be lethal.

### Isolation of mRNA

mRNA was isolated from well fed, synchronized, pre-fertile young adult worms as described previously [50]. For RNAseq experiments, mRNA was collected from six biological replicates. For quantitative real-time RT-PCR experiments, mRNA was collected from at least three biological replicates. The samples used for RNAseq analysis and quantitative real-time RT-PCR were separate, independent samples.

### RNA sequencing

RNA sequencing and analysis was performed as described previously [49]. Six biological replicates per strain were sequenced individually on an Illumina NextSeq 500 sequencer. Read quality was assessed using FastQC v. 0.11.5 (http://www.bioinformatics.babraham.ac.uk/projects/fastqc/) and one-pass aligned to WBcel235 *C*. *elegans* genome using STAR v. 2.5.2b [76] with default parameters and “—outReadsUnmapped None”. The STAR genome index was generated with the corresponding Ensembl WBcel235 build 89 GTF annotations integrated. Transcript abundances were quantified using the “—quantMode GeneCounts” option enabled during alignment. Differential gene expression analysis was performed using the quasi-likelihood framework in edgeR package v. 3.20.1 [77] in R v. 3.4.1. Gene names, gene IDs, and predicted function annotations were downloaded using the biomaRt package v. 2.34.0.

Venn diagrams of overlapping genes were generated using the free online tool BioVenn: http://www.biovenn.nl/. Heat maps were generated using the free online tool from the Broad Institute Morpheus: (https://software.broadinstitute.org/morpheus/).

### Quantitative real-time RT-PCR

Quantitative real-time RT-PCR (qPCR) was performed by converting mRNA to cDNA using a High-Capacity cDNA Reverse Transcription kit (Life Technologies/Invitrogen) according to the manufacturer’s directions. qPCR was performed using a FastStart Universal SYBR Green kit (Roche) in an AP Biosystems RT-PCR machine [51]. The threshold was set at 1.5 as this value routinely fell within the exponential portion of the graph of relative fluorescence versus cycle number. The amount of each mRNA was calculated as the relative copy number of the gene of interest compared to the relative copy number of *act-3* mRNA, then expressed as a percentage of wild-type. The following primer sequences were used for qPCR:

***sod-3*** (AAAGGAGCTGATGGACACTATTAAGC, AAGTTATCCAGGGAACCGAAGTC)

***dod-3*** (AAGTGCTCCGATTGTTACGC, ACATGAACACCGGCTCATTC)

***mtl-1*** (ATGGCTTGCAAGTGTGACTG, GCTTCTGCTCTGCACAATGA)

***sodh-1*** GAAGGAGCTGGAAGTGTTGTTC, CTCCACGTATAGTGAGGTACTCCTG)

***ftn-1*** (GAGTGGGGAACTGTCCTTGA, CGAATGTACCTGCTCTTCCA)

***acs-17*** (GGAGACTATCACTGGAGAAGCTATG, GAACTGCTTCGTCTCCAAGAGTAG)

***gpd-2*** (CTCCATCGACTACATGGTCTACTTG, AGCTGGGTCTCTTGAGTTGTAGAC)

***icl-1*** (TGTGAAGCCGAGGACTACCT, TCTCCGATCCAAGCTGATCT).

### Nuclear localization of DAF-16

Nuclear localization of DAF-16 was visualized using a reporter strain in which *daf-16* is fused to GFP: TJ356 *zIs356 [Pdaf-16*::*daf-16a/b*:*GFP+rol-6(su1006)]* [24]. Worms were immobilized using levamisole on an unseeded NGM plate prior to visualization using a Nikon SMZ1500 fluorescence dissecting microscope. In the *clk-1* and *isp-1* strains, we performed experiments in a *daf-16* mutant background so that the nuclear localization of the endogenous DAF-16 would not interfere with the nuclear localization of DAF-16:GFP, as others had done previously [35].

### Activation of *Psod-3*::*GFP* reporter strain

A *Psod-3*::*GFP* reporter strain was used to monitor the activation of DAF-16 target genes [34]. Quantification of reporter activity was performed as described previously [28].

### Lifespan

Lifespan assays were completed at 20°C except where noted. Lifespan was measured on NGM plates containing 25μM FUdR except where noted. This concentration inhibits the development of progeny after the first transfer and has minimal effect on longevity compared to NGM plates with no FUdR [78]. Lifespans involved *zIs356* worms were performed on plates containing 100 μM FUdR as these worms do not show extended longevity on NGM plates with no FUdR. For each lifespan assay, three biological replicates of at least 50 worms per replicate were performed. Each replicate was performed by a different experimenter. All of the experimenters were blinded to the genotype and or the treatment (RNAi knockdown) of the samples being tested. Worms that crawled off the plate, had internal hatching of progeny or expulsion of internal organs were censored.

### RNA interference

RNAi was performed on NGM plates containing 1 μg/ml IPTG and 50 μg/ml carbenicillin. A single colony of RNAi bacteria from a freshly streaked LB-Tet-Amp plate was grown for 10–12 hours in LB media containing 50 μg/ml carbenicillin. Bacteria were concentrated 5X prior to seeding plates. Bacteria were allowed to dry and grow for 2 days before introducing worms. Three different paradigms were used to administer RNAi depending on the effect of the gene knockdown on fertility and development: RNAi treatment was begun either at the parental L4 generation, at the egg stage or at the L4 stage of the experimental animals. The paradigm utilized was chosen to allow worms to reproduce and to develop to adulthood. The majority of genes targeted by RNAi were knocked down by the standard L4 parental paradigm (*daf-16* (R13H8.1), *pqm-1* (F40F8.7), *cst-1/2* (F14H12.4 and C24A8.4 both targeted by F14H12.4), and *math-33* (H19N07.2). *imb-2* and *bar-1* knockdown using the L4 parental paradigm resulted in larval arrest, sterility, and/or proclivity to extrude the germline (resulting in high levels of censoring). To circumvent these phenotypes, yet allow strongest RNAi knockdown and assessment of lifespan, the following paradigms were implemented. For *imb-2* (R06A4.4), RNAi treatment was initiated at the egg stage for the experimental animals. *bar-1* (C54D1.6) knockdown was performed by directly plating L4 worms to RNAi plates.

### Dihydroethidium staining for ROS

The levels of ROS were measured using the ROS-sensitive dye dihydroethidium (DHE). Approximately 100 day 1 adult worms were picked into a 1.5 ml microcentrifuge tube and washed 3 times in PBS. On the final wash, the level of PBS was reduced to 100 μl, and 100 μl of 30μM DHE was added. Worms were incubated for 1 hour on a shaker at room temperature, washed 3 times in PBS, mounted on a 1.5% agarose pad and immobilized with 5mM levamisole. 30–40 worms were imaged for 3 biological replicates under the 40x objective of an upright Leica compound fluorescence microscope (DM5500B). Fluorescence intensity of ethidium labeled ROS was quantified in the anterior pharynx using a ROI (region of interest) method and ImageJ.

### Statistical analysis

Statistical significance for lifespan assays was assessed using the log-rank test to compare survival curves using GraphPad Prism Version 5. For comparisons involving more than two genotypes or treatments, we used ANOVA to test the significance of differences followed by a Bonferroni posttest to identify specific differences. Error bars indicate standard error of the mean. To determine which genes from the RNAseq data are significantly modulated, we used the quasi-likelihood framework in edgeR package v. 3.20.1 [77] in R v. 3.4.1. To assess the significance of overlapping genes between two strains we used hypergeometric tests, via R, to determine if the number of overlapping up-regulated or down-regulated genes between the two strains was significantly greater than what would be expected by chance. A Benjamini-Hochberg false-discovery rate (FDR) adjustment was used to maintain a 5% FDR after multiple testing.

## Supporting information

S1 Fig Increasing ROS induces mild nuclear localization of DAF-16. The subcellular localization of DAF-16 was monitored using *Pdaf-16*::*daf-16*:*GFP* worms. *clk-1* and *isp-1* worms exhibit weak to medium nuclear localization of DAF-16. Similarly, increasing ROS through treatment with 4 mM paraquat for 24 hours or 300 uM juglone for 2 hours causes mild nuclear localization of DAF-16. Treating worms with 35°C heat for 2 hours induces strong nuclear localization of DAF-16.
(TIF)

S2 Fig Lifespan of *daf-16* mutants is dependent on experimental conditions. To explore the effects of experimental conditions on *daf-16(mu86)* lifespan we varied temperature and FUdR concentration. **A,B.** We found that at 20°C, *daf-16(mu86)* lifespan was similar to wild-type independent of FUdR concentration. **C.** At an elevated temperature of 25°C, *daf-16(mu86)* worms show markedly decreased lifespan. Under conditions in which the *daf-16(mu86)* mutation only mildly decreases lifespan in wild-type worms (20°C, 100 μM FUdR), this mutation markedly decreases longevity in *clk-1* (**D**) and *isp-1* (**E**) mutants. P-values for D,E indicate the significance between red and purple lines. Data and N for the lifespan experiments are included in **S2 Table**.
(TIF)

S3 Fig The ability of mitochondrial mutations *clk-1*, *isp-1* and *nuo-6* to increase lifespan is diminished by decreased levels of DAF-16. The increase in mean (**A**) and maximum (**B**) lifespan that results from mutations in *clk-1*, *isp-1* or *nuo-6* is less when *daf-16* levels are reduced using RNAi. Similarly, the increase in mean (**C**) and maximum (**D**) lifespan that results from mutations in *clk-1* and *isp-1* is less in the presence of the *daf-16(mu86)* deletion mutation. Values for individual replicates are shown in the table below. Error bars indicate SEM. *p<0.05, **p<0.01, ***p<0.001.
(TIF)

S4 Fig Overexpression of DAF-16 increases lifespan in wild-type and mitochondrial mutant worms. The effect of DAF-16 overexpression of lifespan was examined by crossing worms to *zcIs356[Pdaf-16*::*daf-16*:*GFP]* transgenic worms. Lifespans were performed under conditions in which *Pdaf-16*::*daf-16*:*GFP* are long-lived (plates containing 100 μM FUdR). **A.** *Pdaf-16*::*daf-16*:*GFP* worms lived longer than wild-type worms, even in a *daf-16(mu86)* mutant background. The long lifespan of *clk-1* (**B**), *isp-1* (**C**), and *nuo-6* (**D**) worms was all significantly increased by expression of DAF-16:GFP. In contrast, DAF-16:GFP expression had little effect on *daf-2* longevity (**E**). The increase in lifespan resulting from the DAF-16:GFP transgene exhibits an inverse relationship with DAF-16 target gene modulation in the control strain. Error bars indicate SEM. Data and N for the lifespan experiments are included in **S2 Table**.
(TIF)

S5 Fig Increasing mitochondrial superoxide through deletion of *sod-2* activates DAF-16 target genes. **A.** Of the genes that are upregulated in *sod-2* mutants, 36% are upregulated in *daf-2* worms. Percentages indicate the percent of all genes upregulated in *sod-2* and *daf-2* worms. **B.** Of the genes that are downregulated in *sod-2* mutants, 24% are also downregulated in *daf-2* worms. Percentages indicate the percent of all genes downregulated in *sod-2* and *daf-2* worms. **C.** Of the top DAF-16 responsive genes from meta-analysis of DAF-16 target genes performed by Tepper *et al*. 2013 that are upregulated in *daf-2* mutants, many are also upregulated in *sod-2* worms. **D.** Of the top DAF-16 responsive genes from Tepper *et al*., 2013 that are downregulated in *daf-2* worms, some are also downregulated in *sod-2* mutants while others are upregulated. mRNA for each strain was isolated from six biological replicates and sequenced individually.
(TIF)

S6 Fig Long-lived mitochondrial mutant worms have increased levels of reactive oxygen species (ROS) that are not dependent on DAF-16. Levels of ROS were measured by staining worms with the ROS-sensitive dye dihydroethidium (DHE). Both *clk-1* and *isp-1* worms show increased DHE fluorescence compared to wild-type worms indicating elevated levels of ROS. Loss of *daf-16* does not decrease ROS levels in *clk-1* or *isp-1* worms indicating that DAF-16 is not required for the elevated ROS levels in these mutants. The loss of *daf-16* resulted in a small increase in ROS levels in *clk-1* and *isp-1* worms but not in wild-type worms. Error bars indicate SEM. *p<0.05.
(TIF)

S7 Fig MATH-33 is required for the long lifespan of *isp-1* and *nuo-6* mutants. A mutation in *math-33* was found to decrease the lifespan of the long-lived mitochondrial mutants *isp-1* and *nuo-6*, but did not decrease the lifespan of wild-type worms. P-value indicates significance of difference between control and *math-33* mutation for the experimental strain. Data and N for the lifespan experiments are included in **S2 Table**.
(TIF)

S8 Fig Requirement of DAF-16 interacting proteins for *daf-2* and *glp-1* lifespan. P-value indicates difference between EV RNAi (red) and gene of interest RNAi (purple) for mutant strain. Data and N for the lifespan experiments are included in **S2 Table**.
(TIF)

S1 Table Differentially expressed genes. Genes that were significantly upregulated or downregulated in *clk-1*, *isp-1*, *nuo-6*, *daf-2* and *sod-2* mutants are listed.
(XLSX)

S2 Table Lifespan data. Individual data points for all lifespan graphs are provided.
(XLSX)
