# Supplementary figures and images for: Activation of DAF-16/FOXO by reactive oxygen species contributes to longevity in long-lived mitochondrial mutants in Caenorhabditis elegans
Source: PLoS Genet. 2018 Mar 9;14(3):e1007268. doi: 10.1371/journal.pgen.1007268 (PMC5862515; doi:10.1371/journal.pgen.1007268)

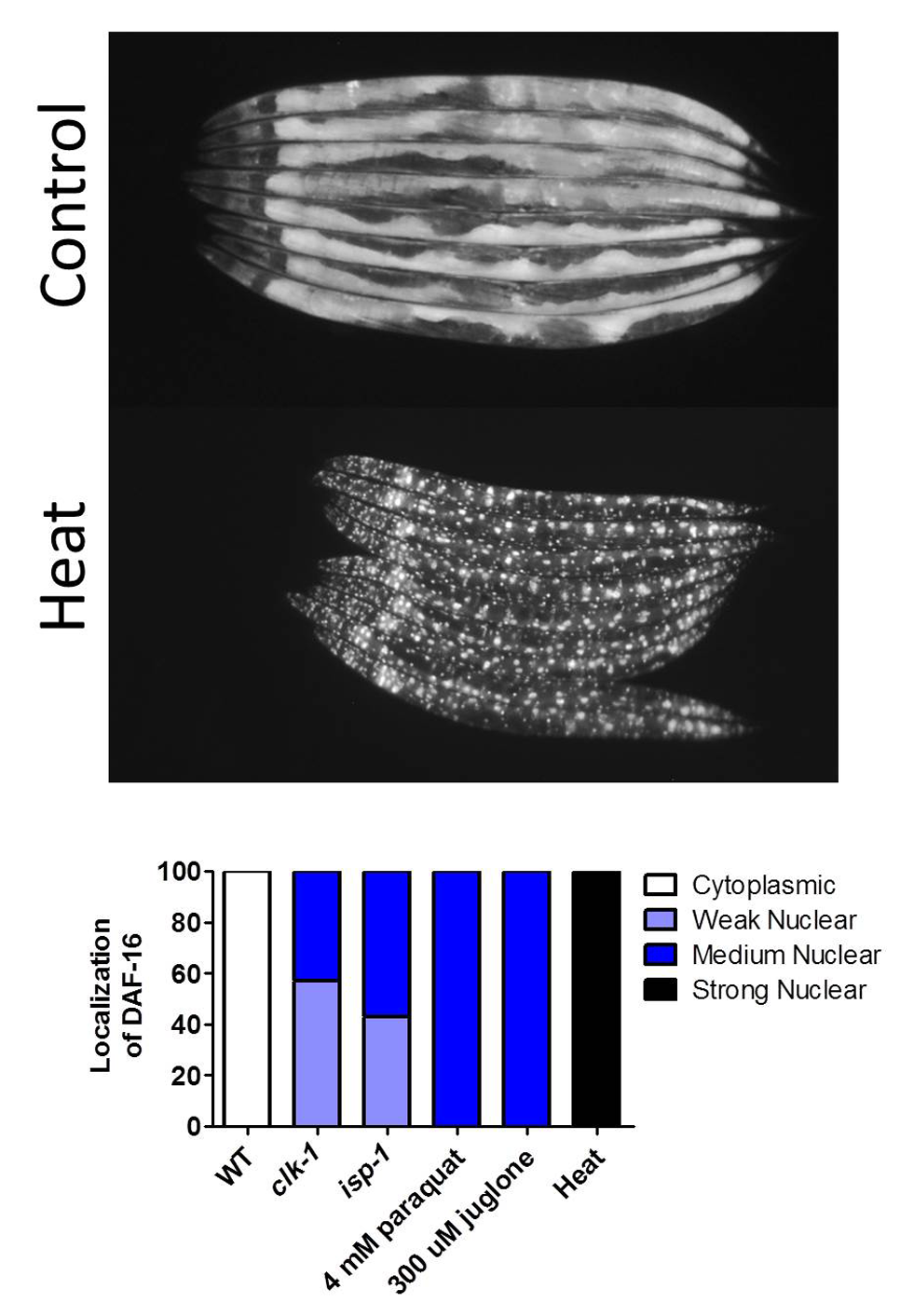

Supplement: S1 Fig — The subcellular localization of DAF-16 was monitored using Pdaf-16::daf-16:GFP worms. clk-1 and isp-1 worms exhibit weak to medium nuclear localization of DAF-16. Similarly, increasing ROS through treatment with 4 mM paraquat for 24 hours or 300 uM juglone for 2 hours causes mild nuclear localization of DAF-16. Treating worms with 35°C heat for 2 hours induces strong nuclear localization of DAF-16. (TIF) [file pgen.1007268.s001.tif]

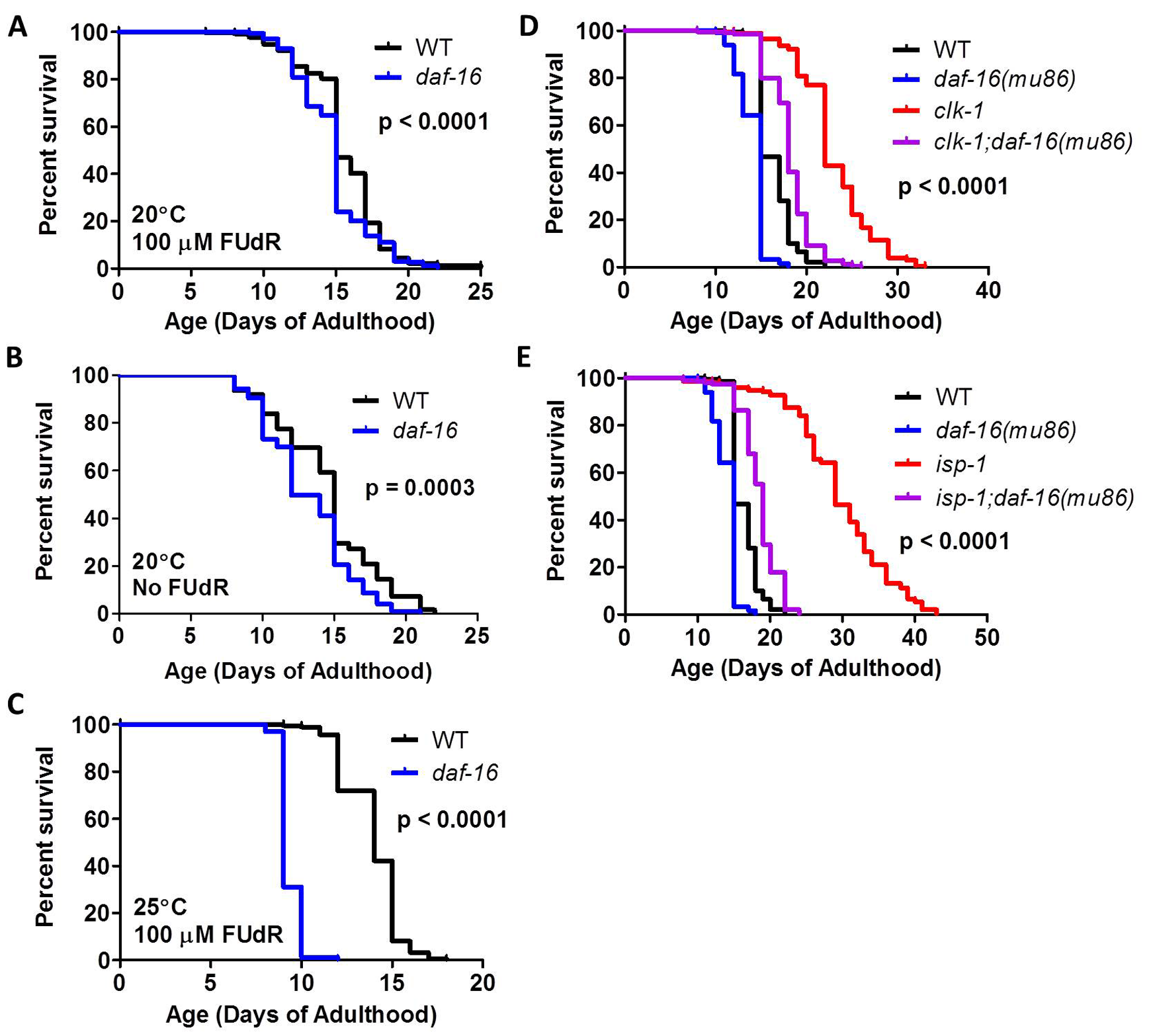

Supplement: S2 Fig — To explore the effects of experimental conditions on daf-16(mu86) lifespan we varied temperature and FUdR concentration. A,B. We found that at 20°C, daf-16(mu86) lifespan was similar to wild-type independent of FUdR concentration. C. At an elevated temperature of 25°C, daf-16(mu86) worms show markedly decreased lifespan. Under conditions in which the daf-16(mu86) mutation only mildly decreases lifespan in wild-type worms (20°C, 100 μM FUdR), this mutation markedly decreases longevity in clk-1 (D) and isp-1 (E) mutants. P-values for D,E indicate the significance between red and purple lines. Data and N for the lifespan experiments are included in S2 Table. (TIF) [file pgen.1007268.s002.tif]

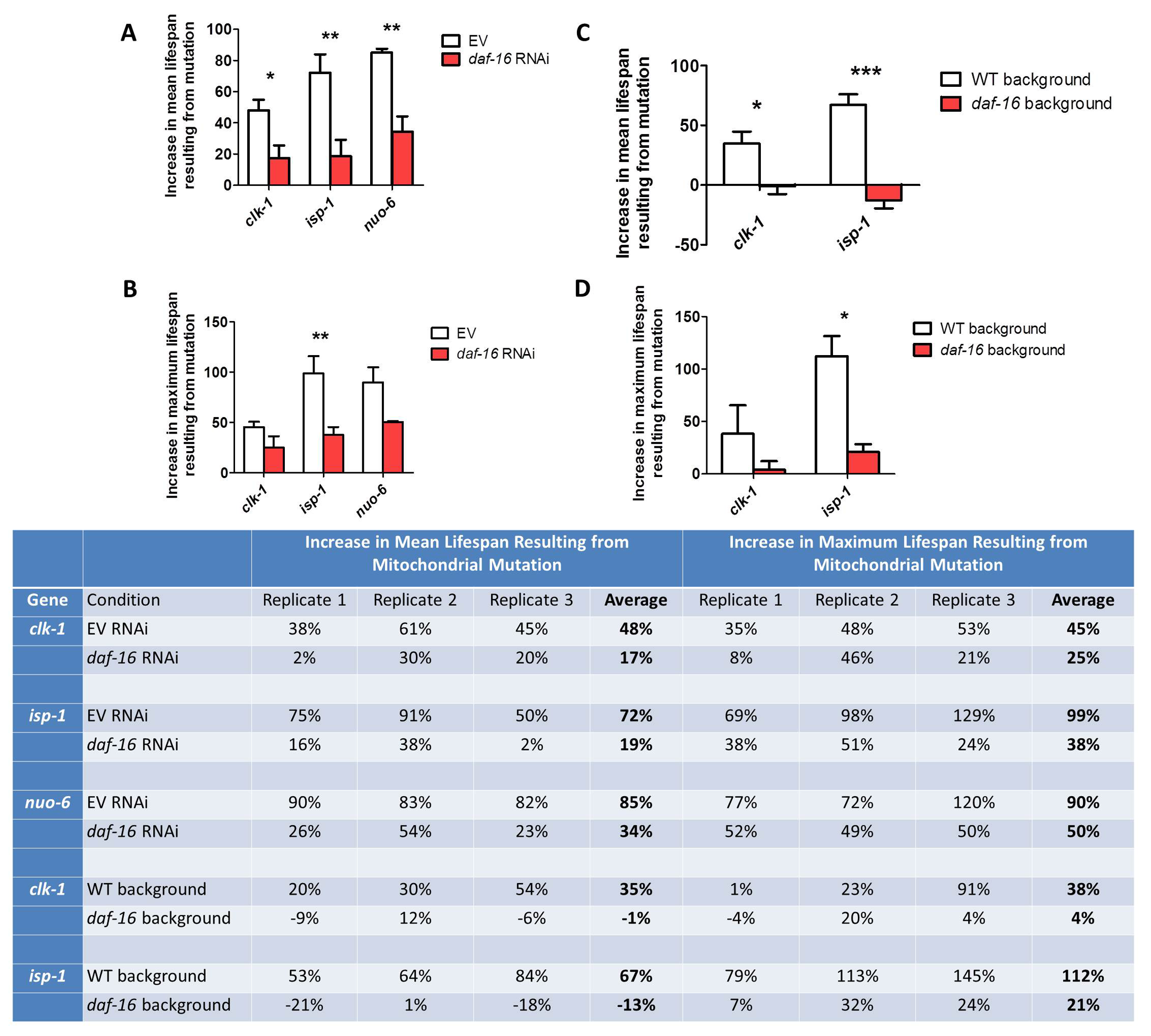

Supplement: S3 Fig — The increase in mean (A) and maximum (B) lifespan that results from mutations in clk-1, isp-1 or nuo-6 is less when daf-16 levels are reduced using RNAi. Similarly, the increase in mean (C) and maximum (D) lifespan that results from mutations in clk-1 and isp-1 is less in the presence of the daf-16(mu86) deletion mutation. Values for individual replicates are shown in the table below. Error bars indicate SEM. *p<0.05, **p<0.01, ***p<0.001. (TIF) [file pgen.1007268.s003.tif]

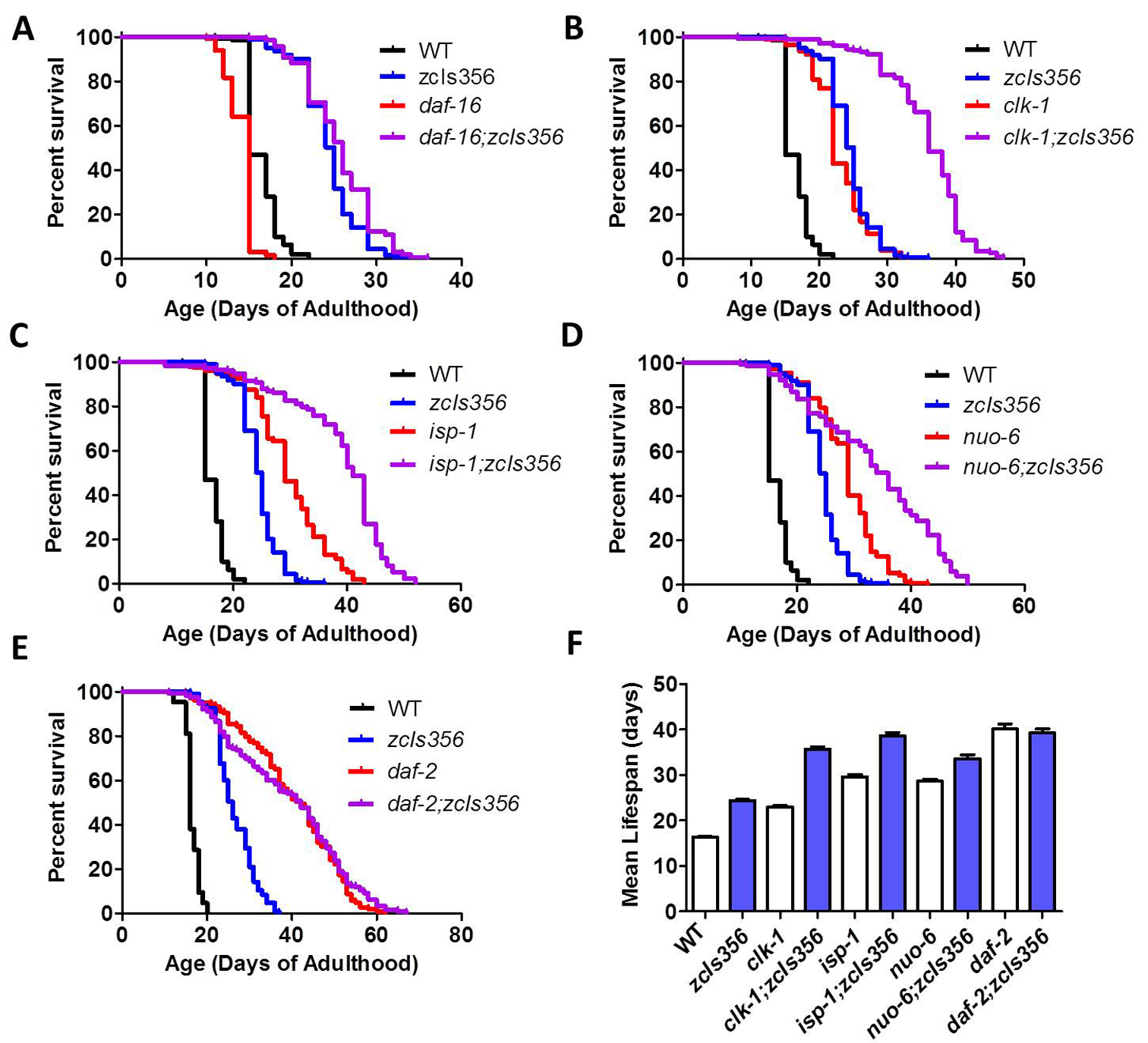

Supplement: S4 Fig — The effect of DAF-16 overexpression of lifespan was examined by crossing worms to zcIs356[Pdaf-16::daf-16:GFP] transgenic worms. Lifespans were performed under conditions in which Pdaf-16::daf-16:GFP are long-lived (plates containing 100 μM FUdR). A. Pdaf-16::daf-16:GFP worms lived longer than wild-type worms, even in a daf-16(mu86) mutant background. The long lifespan of clk-1 (B), isp-1 (C), and nuo-6 (D) worms was all significantly increased by expression of DAF-16:GFP. In contrast, DAF-16:GFP expression had little effect on daf-2 longevity (E). The increase in lifespan resulting from the DAF-16:GFP transgene exhibits an inverse relationship with DAF-16 target gene modulation in the control strain. Error bars indicate SEM. Data and N for the lifespan experiments are included in S2 Table. (TIF) [file pgen.1007268.s004.tif]

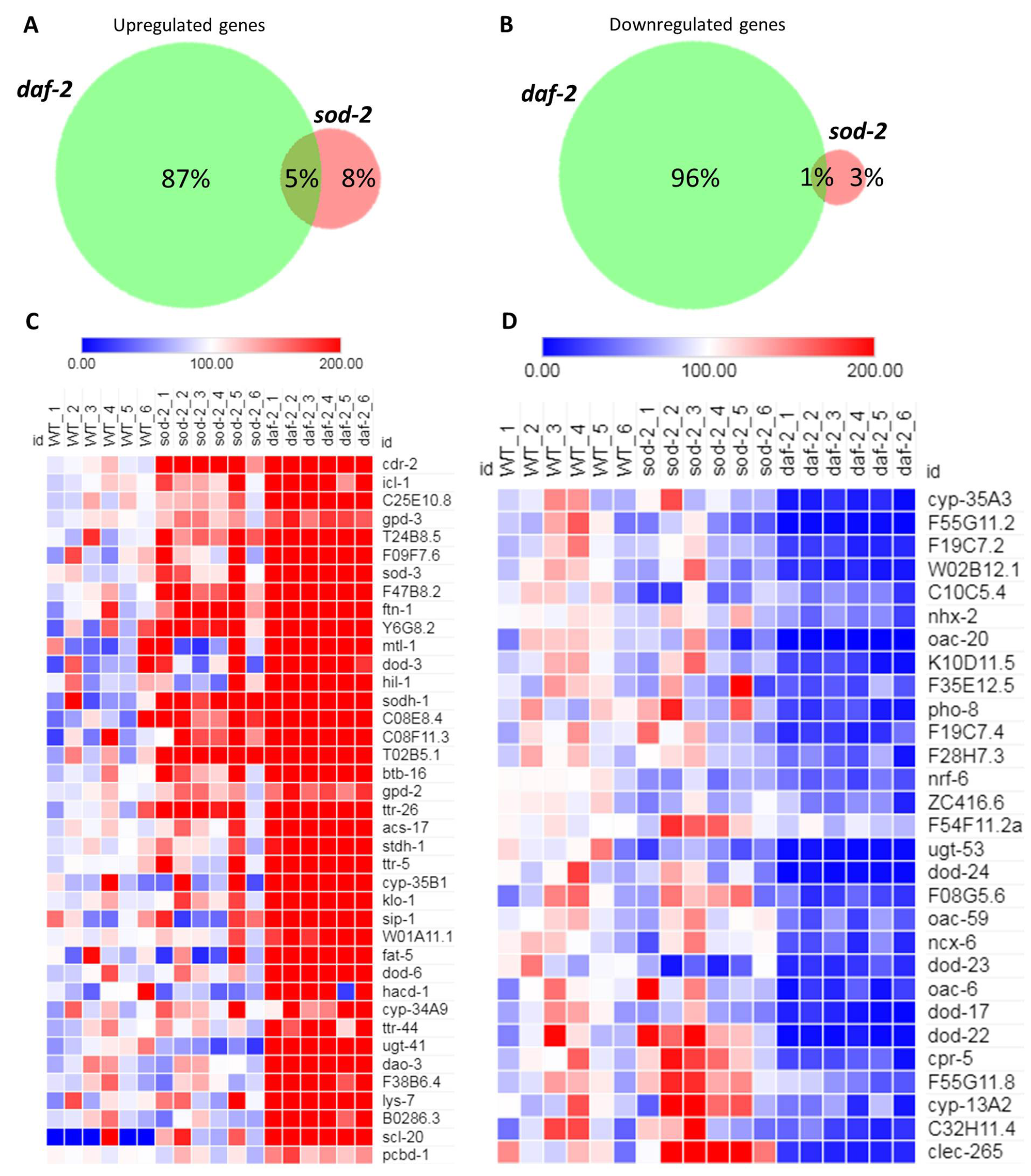

Supplement: S5 Fig — A. Of the genes that are upregulated in sod-2 mutants, 36% are upregulated in daf-2 worms. Percentages indicate the percent of all genes upregulated in sod-2 and daf-2 worms. B. Of the genes that are downregulated in sod-2 mutants, 24% are also downregulated in daf-2 worms. Percentages indicate the percent of all genes downregulated in sod-2 and daf-2 worms. C. Of the top DAF-16 responsive genes from meta-analysis of DAF-16 target genes performed by Tepper et al. 2013 that are upregulated in daf-2 mutants, many are also upregulated in sod-2 worms. D. Of the top DAF-16 responsive genes from Tepper et al., 2013 that are downregulated in daf-2 worms, some are also downregulated in sod-2 mutants while others are upregulated. mRNA for each strain was isolated from six biological replicates and sequenced individually. (TIF) [file pgen.1007268.s005.tif]

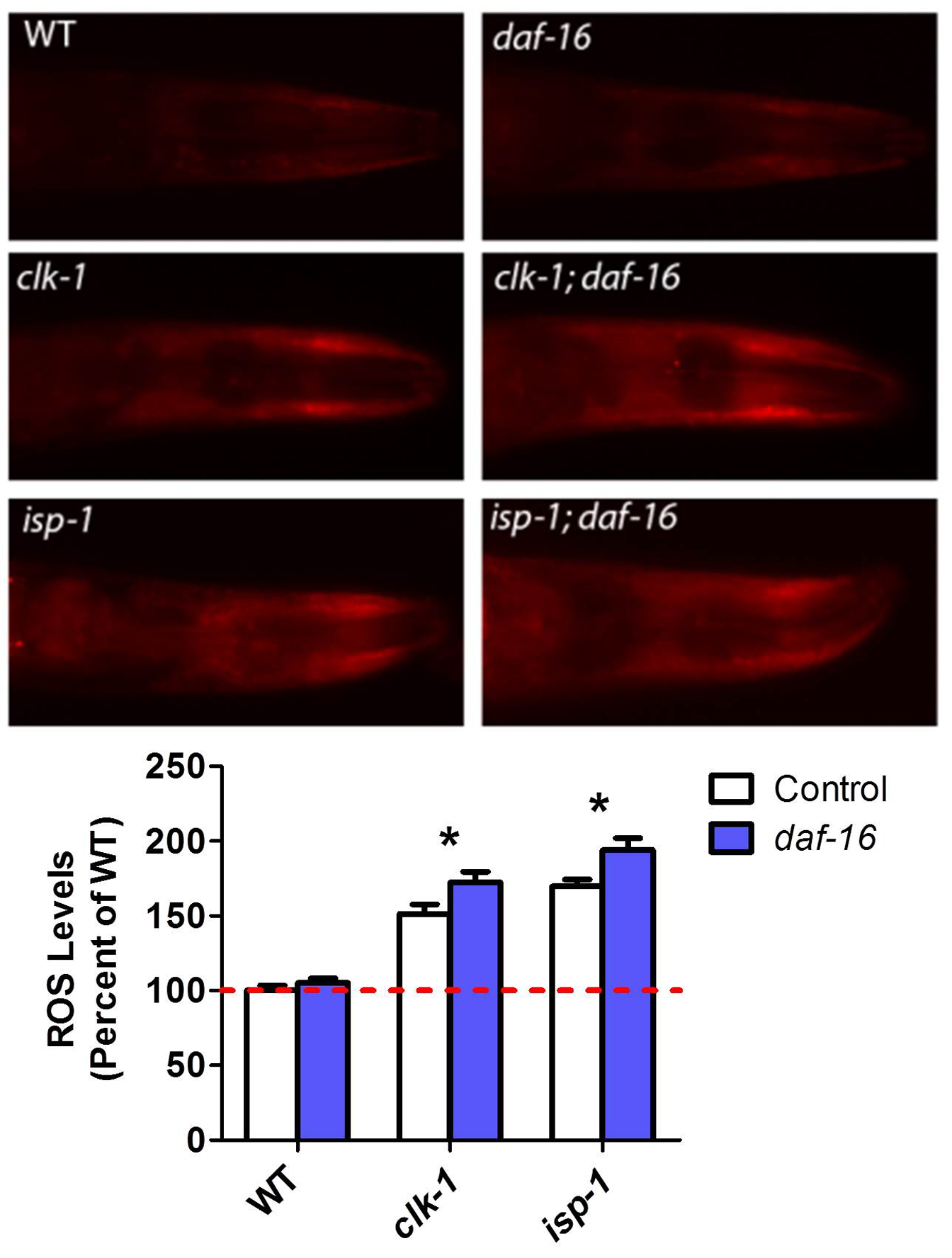

Supplement: S6 Fig — Levels of ROS were measured by staining worms with the ROS-sensitive dye dihydroethidium (DHE). Both clk-1 and isp-1 worms show increased DHE fluorescence compared to wild-type worms indicating elevated levels of ROS. Loss of daf-16 does not decrease ROS levels in clk-1 or isp-1 worms indicating that DAF-16 is not required for the elevated ROS levels in these mutants. The loss of daf-16 resulted in a small increase in ROS levels in clk-1 and isp-1 worms but not in wild-type worms. Error bars indicate SEM. *p<0.05. (TIF) [file pgen.1007268.s006.tif]

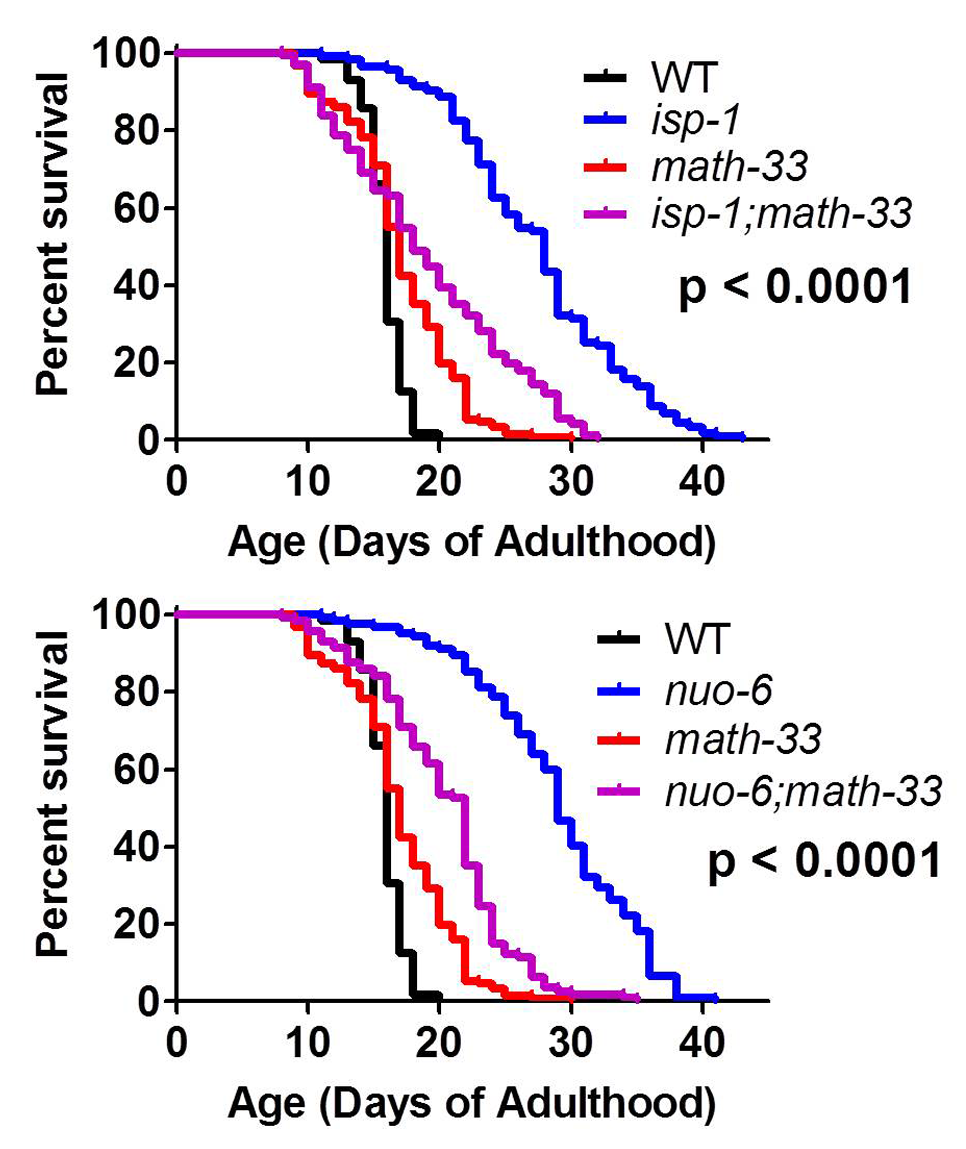

Supplement: S7 Fig — A mutation in math-33 was found to decrease the lifespan of the long-lived mitochondrial mutants isp-1 and nuo-6, but did not decrease the lifespan of wild-type worms. P-value indicates significance of difference between control and math-33 mutation for the experimental strain. Data and N for the lifespan experiments are included in S2 Table. (TIF) [file pgen.1007268.s007.tif]

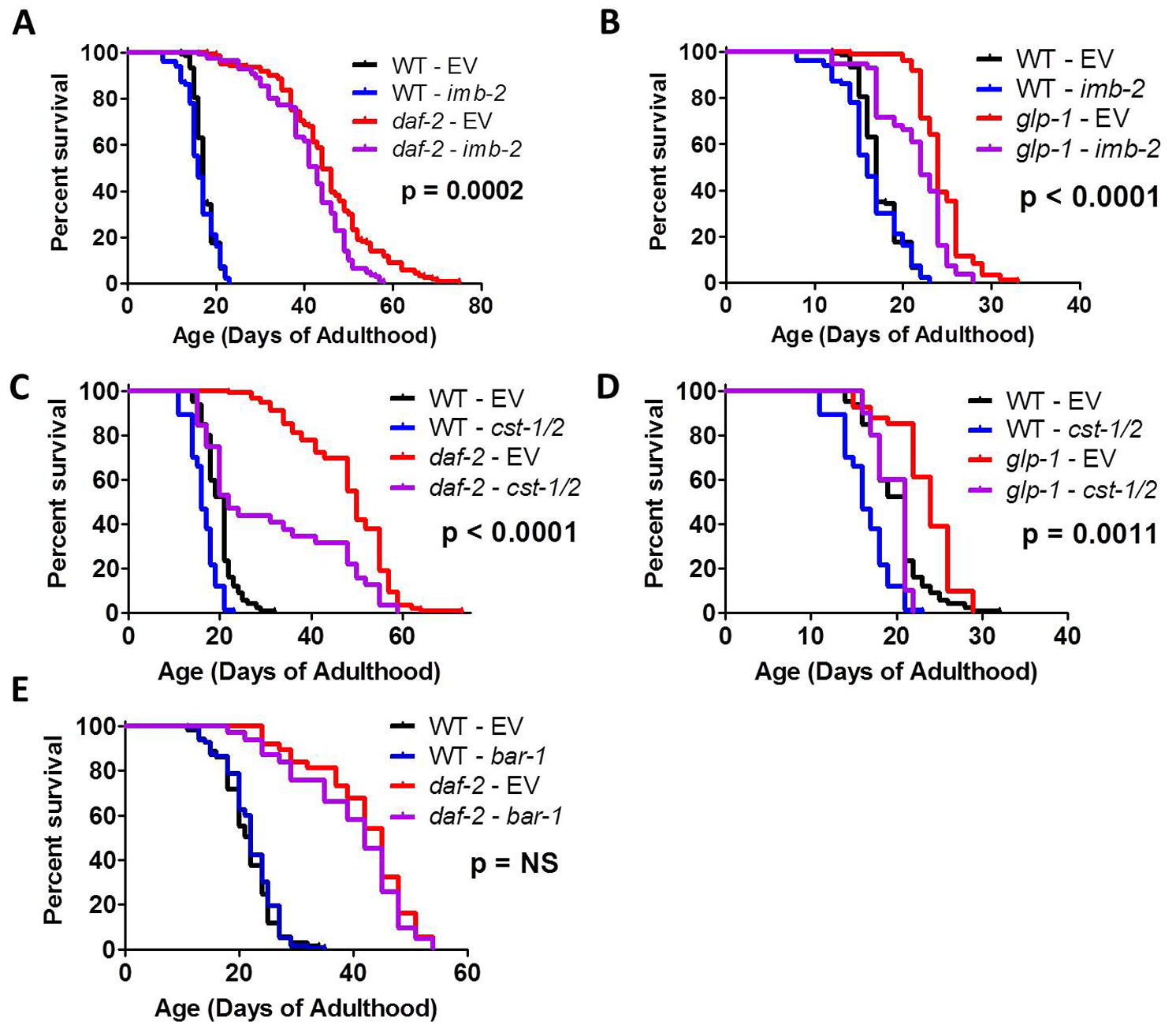

Supplement: S8 Fig — P-value indicates difference between EV RNAi (red) and gene of interest RNAi (purple) for mutant strain. Data and N for the lifespan experiments are included in S2 Table. (TIF) [file pgen.1007268.s008.tif]
